# Supplementary material for: Knowledge integration and decision support for accelerated discovery of antibiotic resistance genes
Source: Nat Commun. 2022 Apr 29;13:2360. doi: 10.1038/s41467-022-29993-z (PMC9055065; doi:10.1038/s41467-022-29993-z)
Supplement: Supplementary file 1 — Supplementary Information [file 41467_2022_29993_MOESM1_ESM.pdf]

Knowledge Integration and Decision Support for Accelerated Discovery of Antibiotic Resistance Genes

Jason Youn[1,2,3], Navneet Rai[1,2,3], and Ilias Tagkopoulos[1,2,3*]

[1]Department of Computer Science, University of California at Davis

[2]Genome Center, University of California at Davis

[3]USDA/NSF AI Institute for Next Generation Food Systems (AIFS)

[*]itagkopoulos@ucdavis.edu

SUPPLEMENTARY INFORMATION

Table of Contents

1 Supplementary Text 4

1.1 Knowledge Graph Constructor 4

1.1.1 Data collection 4

1.1.2 Knowledge inference 8

1.1.3 Analysis of the knowledge graph 9

1.2 Inconsistency Resolver 9

1.2.1 Resolution algorithms 10

1.2.2 Evaluation of the algorithms using a synthetic dataset 11

1.2.3 Inconsistency resolution results 14

1.3 Hypothesis Generator 16

1.3.1 Preprocessing the knowledge graph 17

1.3.2 Path Ranking Algorithm (PRA) 19

1.3.3 Multilayer Perceptron (MLP) 21

1.3.4 Stacked 22

1.3.5 Other graph embedding methods 23

1.3.6 State-of-the-art knowledge graph completion methods 24

1.3.7 Optimization and evaluation 24

1.3.8 Hypothesis generation on individual sources 25

1.3.9 Multi-iteration hypotheses generation 26

1.3.10 Hypothesis generation results 27

1.3.11 Consistency of the KIDS-generated hypotheses 27

1.3.12 Wet-lab validation 29

1.3.13 The similarity of previously unknown ARGs to known ARGs 29

1.3.14 Dissemination of previously unknown ARGs across microbial communities 30

1.3.15 Identification of bacteria harboring a maximum number of genes homologous to 6 previously unknown CRA genes 30

1.3.16 Construction of in-frame single-gene knockouts of the *S. enterica* LT2 . 31

2 Supplementary Figures 32

3 Supplementary Tables 48

4 References 63

1 Supplementary Text

1.1 Knowledge Graph Constructor

1.1.1 Data collection

To construct a comprehensive knowledge graph of *E. coli* antibiotic resistance, existing knowledge bases and literature were integrated. A summary of the 10 different sources we used in our work can be found in **Supplementary Table 1**. The following list describes each source in detail.

- CARD[1]: From version 2.0.0 of the Antibiotic Resistance Ontology (ARO), two predicates '*targeted_by_drug*' and '*confers_resistance_to_drug*' for only the *E. coli* genes were extracted. This results in a total of 147 triples between 72 *E. coli* genes as subject and 33 antibiotics as an object, where predicates are renamed from '*targeted_by_drug*' and '*confers_resistance_to_drug*' to '*targeted by*' and '*confers resistance to antibiotic*', respectively.
- Gene Ontology (GO) dataset[2]: From version 2.4.26 of AmiGO 2, we downloaded annotation data for *E. coli* K-12. Note that a part of its knowledge originally comes from external sources like EcoCyc[3], but we still consider them as GO. We represent them using the following three types of triples types: (*gene*, *has*, *molecular_function*), (*gene*, *is part of*, *cellular_component*), and (*gene*, *is involved in*, *biological_function*). This results in 17,739 triples.
- Liu et al.[4]: In this work, among the 3,985 single-gene knockouts (KEIO) in *E. coli*, 283 strains showed susceptibility to 1 of 14 antibiotics. The work then extends to 8 more antibiotics to test the susceptibility of 283 screened strains. From the initial

screening of 14 antibiotics, 3,985 knockout strains without a response of susceptibility to 14 antibiotics are considered as negative results. In the second screening of 283 strains for 8 antibiotics, any strains without a response of susceptibility to 8 antibiotics are considered as negative results. Any genes with positive results are considered to confer intrinsic resistance to a varied set of antibiotics, as their deletion renders the cell more sensitive than the wild type. We curate the facts discovered in this study using the triple types (*gene*, *confers resistance to antibiotic after 18 hours*, *antibiotic*) for the positive results and (*gene*, *confers no resistance to antibiotic after 18 hours*, *antibiotic*) for the negative results. As a result, a total of 55,877 triples are created from this study.

- Tamae et al.[5]: This work, conducted by the same group that published Liu et al.[4], presents strains that are susceptible to 1 of 7 antibiotics among 3,985 single-gene knockouts in *E. coli*. We apply the same representation used in Liu et al. dataset. This process results in 26,926 triples.
- Shaw et al.[6]: This study presents genes with significant fold-change in expression (profiled with gene expression microarrays) 30 minutes after induction of 4 antibiotics (norfloxacin, kanamycin, rifampicin, and ampicillin) at a different drug concentration of each. From this, we collected genes with positive fold-change at the highest drug concentration of each. This results in a total of 145 facts about 139 genes upregulated by 4 antibiotics, and we represent them using the triple type (*gene*, *upregulated by antibiotic after 30 mins*, *antibiotic*). They examined the expression levels of 3,913 genes. Among them, any genes that are not upregulated in this study are represented using the triple type (*gene*, *not*

upregulated by antibiotic after 30 mins, *antibiotic*). This results in a total of 15,327 triples.

- Nichols et al.[7]: A seminal work in chemical genomics in *E. coli* was published in which a library of over 4,000 Keio[8] knockout strains was screened under many different chemical and physical conditions using phenotype microarray. In their work, individual strains were plated robotically in 1,536-well format, and colony size was investigated to determine fitness. We used the published normalized dataset of this raw data. Please note that we took antibiotics with the highest concentration to be conservative on the findings. Statistical testing was performed based on the description of the original article ($FDR < 0.05$), and we only considered statistically significant results in the negative tail of fitness score distribution (*i.e.,* gene deletions that show increased susceptibility to an antibiotic over wild-type). Missing values are imputed with Random Forest before statistical testing. Among them, we only took gene IDs with clear mappings to the original gene symbol. There was a total of 51 antibiotics, and we identified 2,700 pairs of genes and antibiotics that are considered to confer resistance to the antibiotic. We represented those positive findings using the triple type (*gene*, *confers resistance to antibiotic after 15 hours*, *antibiotic*). Any of the genes with no positive findings for the 51 antibiotics are represented using the triple type (*gene*, *confers no resistance to antibiotic after 15 hours*, *antibiotic*). This results in a total of 186,941 triples.
- Zhou et al.[9]: This work measures nearly 2,000 growth phenotypes in Phenotype Microarrays for *E. coli* K-12 mutants with individual deletions of all two-component

systems (a total of 47 genes). Among them, there were a total of 31 antibiotics, and 78 positive findings of 28 genes (*i.e.,* mutants that show increased susceptibility to antibiotics over wild-type) were identified, and we represent them using the triple type (*gene*, *confers resistance to antibiotic after 36 hours*, *antibiotic*). Any of 47 genes with no positive findings for 31 antibiotics are represented using the triple type (*gene*, *confers no resistance to antibiotic after 36 hours*, *antibiotic*). This results in 1,457 triples.

- Soo et al.[10]: This work examined the effect of *E. coli* genes from the ASKA library[11] overexpressed on plasmids challenged by 237 toxic chemicals, among which results for the 44 antibiotics were extracted. In this study, it found genes conferring increased fitness (growth rates) in the presence of toxins compared to control, which we consider them positive findings. A total of 59 positive findings of 32 genes were identified, which we represent using the triple type (*gene*, *confers resistance to antibiotic after 7 days*, *antibiotic*). The rest of the genes with no positive findings of 44 antibiotics are represented using the triple type (*gene*, *confers no resistance to antibiotic after 7 days*, *antibiotic*). This results in 188,936 triples.
- hiTRN[12]: The original hiTRN data has 6,754 gene-regulatory relations with 207 transcription factors (TFs). Among them, 2,159 gene-regulatory relations with 14 TFs were from the ChIP experiments. We include the gene-regulatory relations in the antibiotic resistance knowledge base to use them in training the hypothesis generator. We considered any *E. coli* genes not reported in the ChIP experiments as negative facts with regards to binding with the 14 TFs. This creates negative triple types (*gene*, *no activates*, *gene*) and (*gene*, *no represses*, *gene*). Along with

positive triples types (*gene*, *activates*, *gene*) and (*gene*, *represses*, *gene*), hiTRN data results in a total of 101,878 triples.

- Girgis et al.[13]: The authors exposed a transposon-mutagenized library of *E. coli* to each of 17 antibiotics, propagating the library for multiple generations. Then they determined the quantitative contribution of each gene to *E. coli*'s intrinsic antibiotic susceptibility using a microarray-based genetic foot-printing technique. From their resource, we found a total of 576 positive findings of 430 genes, which can be represented using the triple type (*gene*, *confers resistance to antibiotic after 3 days*, *antibiotic*). After the exclusion of gene-antibiotic pairs with no available data, the rest of the *E. coli* genes with no positive findings of 17 antibiotics are represented using the triple type (*gene*, *confers no resistance to antibiotic after 3 days*, *antibiotic*). This results in 63,636 triples.

In addition to the information provided above, more detailed source characteristics are provided in **Supplementary Table 6**.

1.1.2 Knowledge inference

We manually generated the 15 sets of knowledge inference rules defined in **Supplementary Data 6** after careful inspection of the existing triple types in the knowledge graph. Application of these knowledge inference rules generated 20,841 new triples, therefore increasing the number of total triples in the knowledge graph by 3.16% (from 658,726 to 679,567). However, such a manual approach cannot guarantee complete coverage of all possible rules. We, therefore, considered using an automated approach by utilizing automatic knowledge graph construction methods like COMET[14].

However, we ultimately decided to leave it as future work since more extensive analysis needs to be performed to ensure that such an automated approach does not create unwanted noise in the data, therefore negatively affecting the downstream performance of the hypothesis generators.

1.1.3 Analysis of the knowledge graph

Among the 7,917 nodes in the knowledge graph, 4,488 were *E. coli* genes (55.0%), 1,782 were molecular functions (22.5%), 1,522 were biological processes (19.2%), 152 were cellular components (1.9%), and 104 were antibiotics (1.3%) (**Figure 2D**). We then classified the 104 antibiotics in the knowledge graph into 6 different taxonomic groups using a chemical classification ontology[15] (**Supplementary Figure 3**) and analyzed the distribution of the CRA triples. The results show that the organoheterocyclic compounds group which contains a ring with at least one carbon atom and one non-carbon atom was the most prevalent group containing 28 antibiotics. The CRA triples belonging to this antibiotic group were also the most well-explored ones with 86.02% of the whole data being already covered in the knowledge graph (**Supplementary Figure 4**).

1.2 Inconsistency Resolver

Multiple truth discovery methods have been proposed over the past decade and have been successfully applied in diverse domains. The primary application domain of these methods is conflict resolution between the web sources, where information conveyed in a particular web page conflicts with that of other web pages[16]. In this setting, three popular approaches exist: iterative methods, optimization methods, and probabilistic methods[17,18].

Recently, using a link prediction method to decide truth among conflicts has been proposed[19]. Some notable works in biological sciences include inconsistency repair in *E. coli* gene regulatory network using answer set programming[20] and inconsistency resolution in signal transduction knowledge using integer linear programming[21].

1.2.1 Resolution algorithms

In addition to the AverageLog[22] inconsistency resolution method described in the **Methods**, we tested 5 additional inconsistency resolution algorithms. The first algorithm is Voting[22], where the triple asserted by most sources is selected. Other algorithms are briefly described below.

1.2.1.1 Sums[23]:

$$R^i(s) = \sum_{t \in T_s} B^{i-1}(t) \tag{1}$$

$$B^i(t) = \sum_{s \in S_t} R^i(s) \tag{2}$$

1.2.1.2 AverageLog[22]:

$$R^i(s) = log|T_s| \frac{\sum_{t \in T_s} B^{i-1}(t)}{|T_s|} \tag{3}$$

$$B^i(t) = \sum_{s \in S_t} R^i(s) \tag{4}$$

1.2.1.3 Investment[22]:

$$R^i(s) = \sum_{t \in T_s} B^{i-1}(t) \frac{R^{i-1}(s)}{|T_s| \cdot \sum_{r \in S_t} \frac{R^{i-1}(r)}{|T_r|}} \tag{5}$$

$$B^i(t) = G\left(\sum_{s\in S_t} \frac{R^i(s)}{|T_s|}\right) \tag{6}$$

where $G(x) = x^g$ and $g = 1.2$ as chosen by the author of the method.

1.2.1.4 PooledInvestment[22]:

$$R^i(s) = \sum_{t\in T_s} B^{i-1}(t) \frac{R^{i-1}(s)}{|T_s| \cdot \sum_{r\in S_t} \frac{R^{i-1}(r)}{|T_r|}} \tag{7}$$

$$B^i(t) = H^i(t) \cdot \frac{G\left(H^i(t)\right)}{\sum_{d\in M_t} G\left(H^i(d)\right)} \tag{8}$$

where $H^i(t) = \sum_{s\in S_t} \frac{R^i(s)}{|T_s|}$ and $g = 1.4$ as chosen by the author of the method.

1.2.1.5 TruthFinder[24]:

$$R^i(s) = \frac{\sum_{t\in T_s} B^{i-1}(t)}{|T_s|} \tag{9}$$

$$B^i(t) = 1 - \prod_{s\in S_t} (1 - R^i(s)) \tag{10}$$

where the hyperparameters ρ and γ (not shown here; refer to the original paper[24] for implementation details) were set to 1.8 and 0.8, respectively, based on the empirical study (**Supplementary Table 7**).

1.2.2 Evaluation of the algorithms using a synthetic dataset

We use a synthetically generated dataset to evaluate the inconsistency resolution algorithms in a controlled setting. In this section, we describe how the synthetic dataset was constructed and how the resolution algorithms were evaluated.

1.2.2.1 Construction of the synthetic dataset

We constructed synthetic datasets from the hiTRN[12] dataset where each synthetic dataset consists of triples from multiple sources with a pre-determined error rate for each source. The performance of inconsistency correction methods is measured for each dataset. In a dataset, multiple sources exist where each source is comprised of triples and the size of the source follows the normal distribution of $N(1{,}000, 333)$. We also investigated the impact of the number of triples per source on the accuracy of inconsistency correction (**Supplementary Table 8**). We varied the source size as some of the inconsistency correction methods we compared take it into account. Each source was falsified by replacing the predicate with its negative counterpart (e.g., replace 'no activates' with 'activates') at the specific error rate E, which follows the normal distribution of $N(E, E/3)$ (*i.e.,* certain triples are incorrect, which creates inconsistency when compared to other source data). For the standard deviation of two normal distributions, a mean divided by 3 was selected to sample positive numbers at a 99.9% chance. We iterated this procedure S times, and therefore, creating S sources. That is, a dataset consists of S sources where average of source size is 1,000 and the average source error rate is E. We experimented with varying $E(0.1, 0.2, 0.3, 0.4)$ and $S(3, 5, 7, 9)$ to see how these variables affect the performance of inconsistency correction methods. For each E and S, we created 1,000 datasets to get the statistics of inconsistency correction performance, thus resulting in 16,000 datasets. We verified 1,000 sampled datasets were enough to approximate the true population of the two parameters (**Supplementary Figure 5**). We also had extra experiments by fixing the parameters of E and S, and there

was no significant difference in accuracy across the six methods (**Supplementary Figure 6** ~ **Supplementary Figure 8**).

1.2.2.2 Rules to identify inconsistencies in the synthetic dataset

The following rules were used to detect inconsistencies in the simulated dataset: 1) '*represses'*, '*no represses*' and 2) '*activates*', '*no activates*'. That is, these rules detect conflicts in gene-regulatory relations where a protein (subject) either represses or does not repress expression of a gene (object) and likewise, a protein (subject) either activates or does not activate expression of a gene (object).

1.2.2.3 Performance metric

Accuracy was measured by the number of correctly resolved inconsistencies divided by the number of total inconsistencies. PCC (Pearson's correlation coefficient) was measured between the true and estimated trustworthiness of sources. The true trustworthiness of the source is essentially $1 - \text{error rate}\ (E)$ of the source.

1.2.2.4 Stopping criteria

In our simulated studies, we observed that the mean difference, δ, of trustworthiness between previous iteration and present iteration is rapidly saturated within 10 iterations (**Supplementary Figure 9**). Therefore, the stopping criterion for the iterative inconsistency correction methods was when the number of iterations reaches 10.

1.2.2.5 Evaluation results.

To investigate the feasibility of the computational correction of inconsistencies, we evaluated six algorithms of inconsistency resolution methods using the synthetic datasets created above. **Supplementary Figure 10** shows AverageLog, Investment,

PooledInvestment, and TruthFinder outperform Sums and Voting overall. As expected, the accuracy of inconsistency correction monotonically increases when the number of sources increases, and when the average percentage of error per source decreases. The performance gap across methods becomes more distinguishable as the average percentage of error per source increases. Interestingly, PooledInvestment begins outperforming when the number of sources increases whereas its performance is suboptimal when the number of sources is few (*e.g.,* 3). AverageLog is particularly accurate when the number of sources is a few. This observation is particularly clear when true and estimated source trustworthiness is compared (**Supplementary Figure 11**). Given those major conflicting facts in the *E. coli* antibiotic resistance knowledge base come from two sources, we have decided to use AverageLog.

1.2.3 Inconsistency resolution results

1.2.3.1 Level 1 inconsistency resolution

Using the inconsistency detection criteria discussed in the **Methods**, we initially identified 291 conflicting sets of triples originating from the two sources Liu et al.[4] and Tamae et al.[5] between the two predicates '*confers resistance to antibiotic after 18 hours*' and 'confers no resistance to antibiotic *after 18 hours*' (**Supplementary Figure 12**). Note that these two sources share identical characteristics such as exposure time, parent strain, and media (**Supplementary Table 6**). However, we found that metronidazole, which is a pro-drug and is converted to the active-drug by bacteria only under the anaerobic condition[25,26], was related to 55 sets of inconsistencies. Therefore, we decided to discard these metronidazole-related inconsistencies and only take into account 236 sets of

inconsistencies for any further evaluation. We then applied the AverageLog inconsistency resolution method, which was chosen from experimenting with the synthetic dataset above, to resolve these 236 sets of inconsistencies. When compared with the ground truth wet-lab validation results, our computational resolution results had an F1 score of 0.24 and an accuracy of 0.86. However, performing an inconsistency resolution where only two conflicting sources exist leads to the problem that the belief $B(t)$ of all resolved triples are equal (**Supplementary Data 2**). In other words, triples from the source that have higher trustworthiness $R(s)$ (in our case Tamae et al. with 0.53) were chosen over the triples from the source Liu et al with 0.40. We denote these as level 1 inconsistencies.

1.2.3.2 Level 2 inconsistency resolution

As we have learned using a synthetic dataset that more sources lead to better resolution performance in **Supplementary Figure 10**, we tested to see if increasing the number of conflicting sources would also translate to improved performance in the real-world scenario. To do this, we observed source characteristics (**Supplementary Table 6**) and found that Nichols et al.[7] shares the same source characteristics as Tamae et al. and Liu et al. except for the shorter exposure time of 15 hours instead of 18 hours. Thus, by alleviating the inconsistency detection criteria to treat predicates '*confers (no) resistance to antibiotic after 15 hours*' and '*confers (no) resistance to antibiotic after 18 hours*' equally, a total of 1,096 sets of conflicting triples (which we denote as level 2) were identified as shown in **Supplementary Figure 13**. Among these, we only compared the original subset of 236 sets of inconsistencies with the ground truth wet-lab validation results from level 1. Results show that the F1 score increased by 75% from 0.24 to 0.42 and the accuracy

increased by 6.98% from 0.86 to 0.92 when compared to the level 1 results (**Supplementary Data 2**).

1.2.3.3 Level 3 inconsistency resolution

Supported by experimental proof that more sources indeed lead to a better resolution, we alleviated the inconsistency detection criteria one more level (level 3) by ignoring all source characteristics including the exposure time. This process allowed us to increase the number of conflicting sources to 8, and a total of 2,131 sets of conflicting triples were identified as shown in **Supplementary Figure 14**. Out of these 2,131 sets, we still compared the original 236 sets of inconsistencies that we have validated in level 1. The results show that the F1 score increased to 0.50 and accuracy increased to 0.94, a 108.30% and 9.30% increase, respectively when compared to the level 1 results (**Supplementary Data 2**). Although we found using the simulated datasets that PooledInvestment works the best when there are 8 sources (**Supplementary Figure 11**), AverageLog still performed the best among all the resolution methods.

1.3 Hypothesis Generator

There are multiple approaches when building statistical models over knowledge graphs. In this work, we implemented three types of hypothesis generator models PRA, MLP, Stacked, TransE, and TransD. We refer the interested reader to a review of the various machine learning techniques over knowledge graphs provided by Nickel et. al[19].

1.3.1 Preprocessing the knowledge graph

1.3.1.1 Use of the negative samples

We investigated how to utilize the negative samples when training different models of the hypothesis generator. The first option was to treat both 8 positive and 4 negative predicates (**Supplementary Table 4**) uniquely, but the key issue with this option was that the knowledge graph was now skewed to the negative predicate types since they were the majority of the edges. Next, we considered treating the negative samples as known negatives of their positive counterparts. For example, we could optimize against the cross-entropy loss when training the MLP using these known negatives. However, since there only existed known negatives for 4 of the 8 predicates (**Supplementary Table 4**), it was not clear how we should train the remaining predicate types. Ultimately, we decided to use alternate methods for training the hypothesis generator models. For the PRA, we decided to follow the original approach used in the paper of choosing the negative samples via the closed-world assumption. When the PRA leverages the closed-world assumption, it chooses the negative samples based on a selection and filtering strategy that helps to identify important samples to train on. For the MLP, we used the more standard training regimen of margin-based ranking loss which generates negatives through corruption (see **Methods**). For the Stacked, we did leverage the negative samples during training, since we trained the ensemble only on edges that consisted of the CRA predicate. This was possible as a separate stacked ensemble is produced for each predicate type in the knowledge graph, and we were only predicting on the CRA predicate. Having said this, we never came to a concrete conclusion on whether using

these negative samples could be beneficial. We believe there is still a potential opportunity in using these negatives during training.

1.3.1.2 Data split for the 5-fold cross-validation

We split the knowledge graph into 5-folds to train/evaluate different hypothesis generator models. As for the distribution of the positive samples, we allotted 72% of the positive CRA triples to the training set, 8% to the validation set used to identify optimal thresholds for the models, and the rest 20% to the test set. The remaining positive samples (non-CRA triples) were then distributed across the training set. As for the distribution of negative samples used for evaluation of the hypothesis generator models, for every positive CRA triple in the knowledge graph, we sampled 49 negatives with the same antibiotic from our known negatives[27,28]. We chose to have this uneven balance of negatives to positive samples to reflect the fact that a gene is far more likely to not confer resistance to a certain antibiotic. Since for some antibiotics, there would not be enough genes to produce negatives for a given edge, we limited the number of negative samples to 49. Having this ratio of negative to positive samples, the baseline average precision can be approximated to 2% when evaluating the performance of our models[29]. Note that in some cases, there were not enough known negatives for an edge to produce negative samples. In such cases, we used the local closed-world assumption to generate synthetic negatives. To construct these negatives for every positive edge that did not have enough negatives, we randomly replaced the gene that was not already a known negative or known positive.

1.3.1.3 Removal of temporal information

As discussed in the **Methods** section of the main manuscript, we removed the temporal information from some of the predicates in the knowledge graph (**Supplementary Table 2** and **Supplementary Table 4**). This decision was to handle the lack of training data if we were to treat each predicate with varying temporal information distinct (e.g., the positive predicate ‘CRA after 7 days’ only has 59 triples). However, removing the temporal information has the side effect of creating potential inconsistencies. For example, although the two triples (*cydX*, CRA after 15 hours, Vancomycin) and (*cydX*, ¬CRA after 18 hours, Vancomycin) supported by Nichols et al.[7] and Tamae et al.[5], respectively, are not inconsistencies in their original form, they become inconsistencies after removing the temporal information. As described in the **Supplementary Information Section 1.2.3.3**, removing the temporal information from the knowledge graph results in an increase of inconsistencies from 236 (level 1 inconsistency) to 2,131 (level 3 inconsistency).

1.3.2 Path Ranking Algorithm (PRA)

1.3.2.1 An observable graph feature model

Observable graph feature models extract features from the observed edges over the knowledge graph to predict the existence of a new edge, and the PRA[30,31] is an example of such a model. Among others, the PRA has been used for link prediction over the Nell knowledge graph[32] and the Knowledge Vault project[27]. Liekens et al.[28] also used a graph feature model like PageRank to predict genes causing disease. The advantage of this type of approach is that the features are readily observable over the graph, therefore

translating to useful reasons why the prediction was made. The models in this category are well suited for modeling local patterns in the data.

The PRA performs random walks over the graph at a bounded step size to identify the existence of new edges over the graph. The features of this model are the path probabilities of reaching an object entity from a subject entity. The PRA leverages the closed-world assumption to identify the negative training samples for the model. As the random walks are performed, paths will also result in the wrong object entities. These subject-object pairs can act as negative samples. The paths generated for each wrong object entity are scored against an untrained model using default initialized weights to rank these negative samples. A selection strategy is then used to choose which negative samples to train for the model. In our case, we chose to use all negative samples found. We leveraged the original Java implementation by the author of the PRA.

1.3.2.2 The path features obtained by the PRA

One advantage of the PRA is that it provides interpretable results. **Supplementary Table 9** and **Supplementary Table 10** show the path features and their corresponding weights trained by the PRA when generating the first and second iteration of hypotheses, respectively. In both iterations, the most important feature identified by the PRA was as follows:

$$gene \xrightarrow{is\ involved\ in} biological\_process \xrightarrow{is\ involved\ in^{-1}} gene \xrightarrow{confers\ resistance\ to\ antibiotic} antibiotic.$$

This path corresponds to a sequence of three predicates linking the gene-antibiotic pair. The inverse sign indicates the inverse direction that the random walker took in creating a

path. In other words, this path tells us that at least one other gene that is involved in the same biological process also confers resistance to the antibiotic of interest. One can take these paths as evidence behind the predictions.

1.3.3 Multilayer Perceptron (MLP)

1.3.3.1 Latent feature model

Another popular approach to this field of research is to generate latent features by using embeddings for the entities and/or predicates in the knowledge graph. The features generated from these types of models are called “latent” because they are not directly observable over the graph. Moreover, these types of relational models are well suited at modeling global patterns that exist over the graph[19]. To produce these latent features, the entities in the knowledge graph are converted to numerical vectors or embeddings that are treated as learnable parameters by the model. The relationship between these entities is then derived from the interaction of their latent features in the respective model. The outputs of these models consist of a single score or confidence indicating whether an edge should exist between the two entities.

For instance, Ding et. al[33] generated latent features by using the Neural Tensor Network[34] to perform event-driven stock market prediction. The Entity-Relation Multilayered Perceptron (ER-MLP)[27], which has been shown to provide comparable results to the Neural Tensor Network while using significantly fewer parameters, was used to predict new facts over the Freebase knowledge graph for the Knowledge Vault project. More recently, the use of holographic embeddings has shown promising results[35].

Another class of latent feature models involves predicting the existence of edges over a knowledge graph by measuring the similarity of the vector-spaced entity embeddings. For instance, TransE[36] identifies the score for a certain edge as the distance between the predicate-specific translations of two entity embeddings. The distance can be measured by using Euclidean distance. Although this type of model requires very few parameters, this is with the cost of modeling performance. Hence, the TransH[37] and TransR[38] have been introduced to improve on this limitation by introducing additional parameters to improve the TransE performance.

1.3.3.2 Word embeddings form clusters based on their entity types

The MLP concatenates a single predicate embedding of size 50 and two entity embeddings of size 50 each to train the model. These embeddings consist of learnable parameters that capture a semantic representation after training. When we reduced the dimensions of the entity embeddings by performing the principal component analysis (PCA)[39], we observed noticeable clusters forming depending on their entity type as shown in **Supplementary Figure 15**. Interestingly, the entity types (*i.e.*, gene, antibiotic, etc.) of these entities were never provided to the MLP during training. It simply learned the entity types on its own.

1.3.4 Stacked

It has been shown experimentally that neither the latent feature model nor the graph feature model can predict optimally on its own[27]. As they are well equipped in modeling different types of patterns in the knowledge graph, researchers have built combined models that incorporate both the global and local perspectives over the knowledge

graph[27,40]. The fused prior models in this category, have shown to have state-of-the-art performance due to this dual nature in pattern recognition. Consequently, we decided to explore this option to automatically generate new hypotheses over the *E. coli* knowledge graph. This ensemble approach using AdaBoost[41] leverages a sequence of one-depth decision trees. Each decision tree is trained on a modified version of the training set. After each iteration of training, when the classifier incorrectly classifies a sample, that sample is upweighted in importance to ensure that the classifier focuses its attention on correcting the mistake during the next iteration. The predictions of each weak learner are combined through a weighted majority vote to make the final prediction.

1.3.5 Other graph embedding methods

We tested graph embedding methods TransE[36] and TransD[42] that model relationships between the entities by interpreting them as a translational operation. That is, the model optimizes the embeddings by enforcing the vector operation of the subject entity embedding plus the relation embedding to be close to the object entity embeddings. We used self-adversarial negative sampling with temperature fixed to 1.0, optimized using Adam[43], fine-tuned the hyperparameters on the validation dataset, and performed early stopping. The range of the grid-search used for hyperparameter search was as follows: negative samples $n \in \{25, 50, 100\}$, embedding dimension $d \in \{128, 256, 512, 1024\}$, margin $\gamma \in \{6.0, 12.0, 24.0\}$, and learning rate $\alpha \in \{0.001, 0.0001\}$. We used the open-source implementation of these models using the OpenKE toolkit[44]. For TransE, the best hyperparameters obtained were $n = 100$, $d = 256$, $\gamma = 12.0$, and $\alpha = 0.001$. For TransD, the best hyperparameters obtained were $n = 100$, $d = 256$, $\gamma = 24.0$, and $\alpha = 0.0001$.

Results of these models are provided in **Supplementary Table 5**. We also tested two additional graph embedding methods SimplE[45] and RotatE[46], but we were not able to find optimal set of hyperparameters that performs better than the PRA even after an extensive grid-search.

1.3.6 State-of-the-art knowledge graph completion methods

In addition to the five methods PRA, MLP, Stacked, TransE, and TransD considered in this work, we also tested more recent state-of-the-art methods that were introduced after the project was conceived. We tested factorization-based knowledge graph completion methods TuckER[47] and performed the hyperparameter search among the following combinations with the best setting marked in bold: learning rate ∈ {**0.0002**, 0.0005, 0.001}, decay rate ∈ {0.99, 0.995, **1.0**}, entity embedding dimension ∈ {**200**}, relation embedding dimension ∈ {**30**, 200}, input dropout ∈ {**0.2**, 0.3}, first hidden dropout ∈ {0.1, 0.2, **0.4**}, second hidden dropout ∈ {0.2, 0.3, **0.5**}, and label smoothing ∈ {0.0, **0.1**}. We used a batch size of 128 and trained for 500 iterations with early stopping. As shown in **Supplementary Table 5**, Tucker has a 0.7% higher F1 score than the stacked model (30.1% vs. 30.8%; *p*-value: 0.65). For our future work, we expect to see higher discovery rates using such state-of-the-art knowledge graph completion methods.

1.3.7 Optimization and evaluation

1.3.7.1 Optimization criteria

A validation set was used to identify optimal thresholds for the PRA, MLP, TransE, TransD, and TuckER. We optimized using the F1-score. Additionally, since we were training new PRA and MLP models for each fold, we optimized the number of classifiers and the

learning rate for the Stacked model during each fold. For this case, we optimized using Average Precision.

1.3.7.2 ROC curve for evaluation

The receiver operating characteristic (ROC) considers the true negatives during evaluation. This metric contains information about how well our model was able to correctly identify a negative sample. Due to this attribute, the ROC is not an ideal metric to use for a highly unbalanced dataset like ours, where the number of positive test samples is significantly less than that of negative samples. Since this true negative metric is relatively unimportant to the positive samples, we produced precision-recall (PR) curves for each model. This metric only considers true positives, false positives, and false negatives. This is a somewhat harder metric since there are significantly fewer positive test samples than negatives. The PR curve has also been shown to provide a more informative metric for retrieval tasks when compared to the ROC curve[48].

1.3.8 Hypothesis generation on individual sources

We wanted to test if the hypothesis generator trained using our knowledge graph predicts better associations than the ones trained using individual sources. To do this, we treated each source as a unique knowledge graph to evaluate the three hypothesis generator models PRA, MLP, and stacked using 5-fold cross-validation (**Supplementary Table 11**). However, we were not able to train PRA on any single source knowledge graph as PRA requires at least two unique predicates to build path features. As we were not able to train the PRA on any single source knowledge graph, we also could not train the stacked model. For the MLP, we were able to get results for 5 sources. The combined AUCPR of the MLP

from these 5 individual sources was 0.26±0.15. This was greater than the AUCPR of the MLP trained on our knowledge graph (0.22±0.01), although statistically insignificant (*p*-value: 0.61).

1.3.9 Multi-iteration hypotheses generation

For the first iteration, we generated 108,078 CRA hypotheses which account for 23.2% of all gene-antibiotic pairs in the knowledge graph (466,752 total possible pairs from 4,488 *E. coli* genes and 104 antibiotics). We then grouped the hypotheses into 5 bins (**Figure 5A**) and tested all hypotheses above 20% probability that we have antibiotics in stock (105 hypotheses out of 149 hypotheses). For the hypotheses with a probability ≤ 20%, we randomly selected 121 hypotheses that we have antibiotics in stock out of 107,929 hypotheses. We observe that 99.9% of the hypotheses (107,929 out of 108,078) belong to the lowest bin (≤ 20%) due to the tendency of genes to not confer resistance to antibiotics. In total, we validated 226 CRA hypotheses with varying probability among which 64 were validated as positives in the first iteration.

For the second iteration, we merged all 226 CRA hypotheses that we validated in the first iteration to expand the knowledge graph. This increased the size of the knowledge graph from 651,758 to 651,984. After training the hypothesis generator again using this updated knowledge graph, we generated 107,852 CRA hypotheses. We then followed the same validation process as discussed for the first iteration. In addition to binning the probability of the generated hypotheses from the two iterations into 5 bins (**Figure 5A**), **Supplementary Figure 16** shows the same analysis but with 10 bins.

1.3.10 Hypothesis generation results

From these two iterations of hypotheses generation, we computationally predicted and experimentally validated a total of 93 CRA hypotheses for 83 *E. coli* genes that confer resistance to one or more of 15 antibiotics (**Figure 5E**). Among these 93 CRA hypotheses, we found that 61 CRA hypotheses were inconsistencies that we did not merge during the inconsistency resolution process (**Supplementary Data 4**). In other words, we only validated and merged 236 inconsistencies out of 2,131 inconsistencies identified from level 3 (see **Section 1.2.3.3**). As we did not merge the remaining 1,895 inconsistencies into the knowledge graph, the hypothesis generator generated hypotheses on these 1,895 inconsistencies. In future work, we expect to address this issue and produce better results.

1.3.11 Consistency of the KIDS-generated hypotheses

We tested the consistency of the hypotheses generated by KIDS using two metrics Kendall's tau[49] and rank-biased overlap (RBO)[50] that checks if two ranked lists are in agreement. Kendall's tau, a widely used correlation-based method, ranges between -1 and +1, where -1 denotes the complete disagreement and +1 denotes the complete agreement between the two ranked lists. However, some of its properties render its application to the KIDS-generated hypotheses less appropriate. For example, Kendall's tau requires two ranked lists to be of the same length, yet the number of hypotheses with probability > 0.20 changes for every run of hypotheses generation due to the stochastic nature of the hypotheses generation models. Moreover, it assigns equal weight to all the items in the ranked list, treating the agreement at the top of the lists (hypotheses with

higher probability) as important as the agreement at the bottom of the lists (hypotheses with lower probability). RBO is an alternative method whose value ranges between 0 and 1, where 0 means complete disagreement and 1 means a complete agreement between the two ranked lists. In contrast to Kendall's tau, RBO allows comparing two disjoint ranked lists of different lengths as well as putting more emphasis on the agreement at the top of the lists. In this work, we provide results for both metrics.

To this end, in addition to the original first iteration hypotheses (**Supplementary Data 3**), we generated 99 different versions of first iteration hypotheses each with unique random seeds. For Kendall's tau, as the length of the ranked lists needs to be identical, we found the rank of the original 149 original first iteration hypotheses with probability > 0.20 among the 99 versions of the hypotheses. Note that each ranked list contains 149 hypotheses. We then generated $\binom{100}{2} = 4{,}950$ pairs of ranked lists which was used to calculate Kendall's tau statistics. For the baseline, we randomly selected 149 hypotheses from 100 versions of randomly generated hypotheses, which were used to calculate Kendall's tau statistics similarly as above. For RBO, as the length of the ranked lists does not need to be identical, we used a more straightforward approach of extracting hypotheses with probability > 0.20 from each one of the 100 versions of first iteration hypotheses (including the original first iteration hypotheses). Similar to Kendall's tau approach, we generated $\binom{100}{2} = 4{,}950$ pairs of ranked lists which were used to calculate RBO statistics while setting the hyperparameter *p* to 0.99. For the baseline, we randomly selected varying numbers of hypotheses from 100 versions of randomly generated hypotheses. For example, if the number of hypotheses with probability > 0.20 in one version of the first

iteration hypotheses was x, we also selected x number of hypotheses from the randomly generated hypotheses. The *p*-value between the KIDS-generated statistics (Kendall's tau or RBO) and the randomly generated hypotheses was calculated using the T-test with a two-sided alternative hypothesis. Finally, among the 2,907 that appeared at least once among the 100 different versions of first iteration hypotheses with probability > 0.20, we identified 11 hypotheses that appeared in all 100 different versions from which 10 were validated to be a positive relationship (**Supplementary Data 11**).

1.3.12 Wet-lab validation

We profiled the antibiotic resistance response of 226 single-gene knockout strains of *E. coli* obtained from the Keio collection[8]. Wild-type *E. coli* strain BW25113 was used as a control. For routine culturing, *E. coli* Wild-type cells were grown in LB medium, while knockouts were grown in media supplemented with 50 µg/ml kanamycin unless otherwise mentioned. To profile the antibiotic resistance response, fresh colonies of required strains were transferred to 96 well plates containing 200 µl of LB broth and grown for 8 hours at 37°C in an incubator shaker (BioTek HTX). Later, a fraction of the culture was transferred using a 96-pin replicator to a plate containing LB agar and the different amount of antibiotics. The plate was incubated overnight at 37°C, and the next day absence or presence of colonies was recorded to identify the MICs (**Supplementary Data 8**). All experiments were performed in biological triplicate.

1.3.13 The similarity of previously unknown ARGs to known ARGs

To show how similar the 6 previously unknown ARGs (*ftsP*, *hdfR*, *lrp*, *proV*, *qorB*, and *rbsK*) are at the sequence level to already known ARGs, we downloaded the nucleotide

sequence of the 4,577 ARGs from CARD[1] (version 3.1.4) and performed BLASTN with our 6 ARGs (*https://blast.ncbi.nlm.nih.gov/Blast.*cgi). The highest-ranked matches are the following: *CTX-M-204* of *Klebsiella pneumoniae* for *lrp* (100% identify, E-value=5.9); *CMH-5* of *Enterobacter cloacae* for *rbsK* (88% identify, E-value=0.93); *cprS* of *Pseudomonas aeruginosa PAO1* for *qorB* (84.8% identify, E-value=0.86); *MOX-9* of *Citrobacter freundii* for *hdfR* (100% identify, E-value=0.84); *OXA-541* of *Pseudomonas putida* for *lrp* (91.7% identify, E-value=0.12); *ErmG* of *Bacteroides thetaiotaomicron* for *lrp* (88.9% identify, E-value=1.2). We also tested how similar the genes that we have predicted to confer no resistance using the hypothesis generator (probability range [0.0 and 0.2]) and further validated in the wet-lab to the ARGs in CARD. Out of the 129 genes, we found 11 genes that have a significant E-value (≤ 0.05).

1.3.14 Dissemination of previously unknown ARGs across microbial communities

Using the MGnify service (*http://www.ebi.ac.uk/metagenomics*), we performed a protein sequence search of our 6 previously unknown ARGs using HMMER (*http://hmmer.org*) against their human digestive system microbiome database which contains 94,342 samples with a cutoff E-value set to ≤ 0.05. We found how much dissemination these genes have in the database for 5 ARGs except for proV which ran into a server-side error (**Supplementary Table 12**).**Identification of bacteria harboring a maximum number of genes homologous to 6 previously unknown CRA genes**

We performed nucleotide mega-BLAST (*blast.ncbi.nlm.nih.gov/Blast.cgi*) to identify the genes homologous to 6 previously unknown CRA genes in other bacterial genera. We found that Salmonella spp. had the maximum number of homologous genes. *Salmonella*

enterica had 5 homologs *ftsP*, *lrp*, *proV*, *rbsK*, and *yifA* (*hdfR* in *E. coli*) with >78% similarity in nucleotide sequences, while homolog of *qorB* was not identified in *S. enterica*.

1.3.16 Construction of in-frame single-gene knockouts of the *S. enterica* LT2

We constructed 5 single-gene KO strains of *S. enterica LT2* using λ-red recombinase system as described elsewhere[8]. Briefly, we PCR amplified the kanamycin cassette from Keio-strain JW1869 using 5 sets of specially designed primers containing end sequences of kanamycin cassette and target genes (**Supplementary Table 13**). Electrocompetent *S. enterica* cells harboring pkD46 plasmid, temperature-sensitive and an ampicillin-resistant plasmid expressing λ-red recombinase system, were transformed with individual sets of PCR amplified kanamycin cassette to replace the 5 target genes with kanamycin marker. Cells were selected on an LB agar plate containing kanamycin and later pkD46 was removed from the cells by growing the cells in LB broth at 40°C[8]. MICs of antibiotics cephradine (for *ftsP* and *rbsK* KOs), geneticin (for *lrp* KO), chloramphenicol (for *proV* KO), and hygromycin B (for *yifA* KO) were measured as described in the methods section of the main manuscript. Wild type *S. enterica* was always used as a reference strain (**Supplementary Data 9**).

2 Supplementary Figures

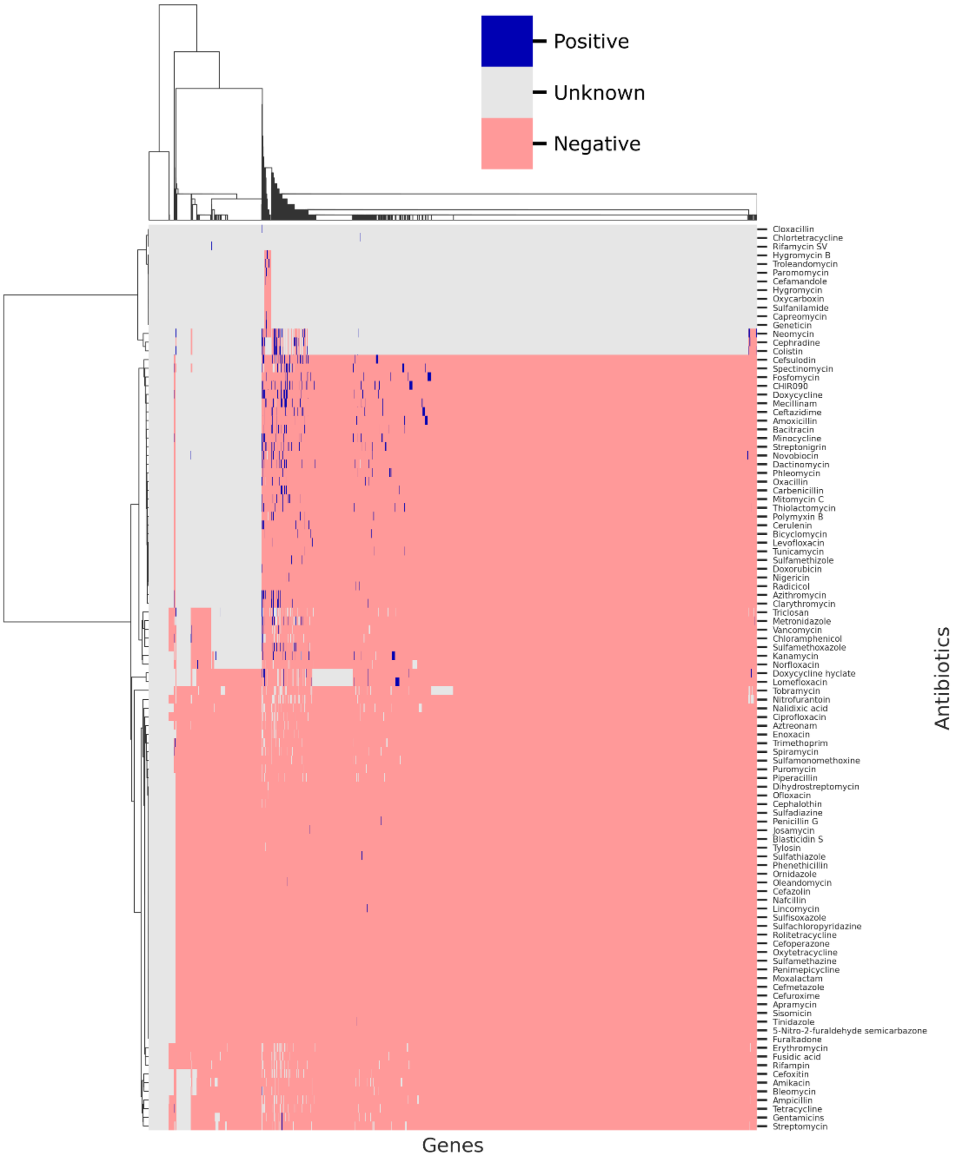

Supplementary Figure 1. Hierarchy-clustered heatmap showing the curation status of all pairwise combinations of genes and antibiotics. Positive (blue) and negative (red) cells denote there exist positive and negative CRA predicate, respectively, between the corresponding genes and antibiotics. Unknown (gray) cells denote possible candidates for either positive or negative CRA association.

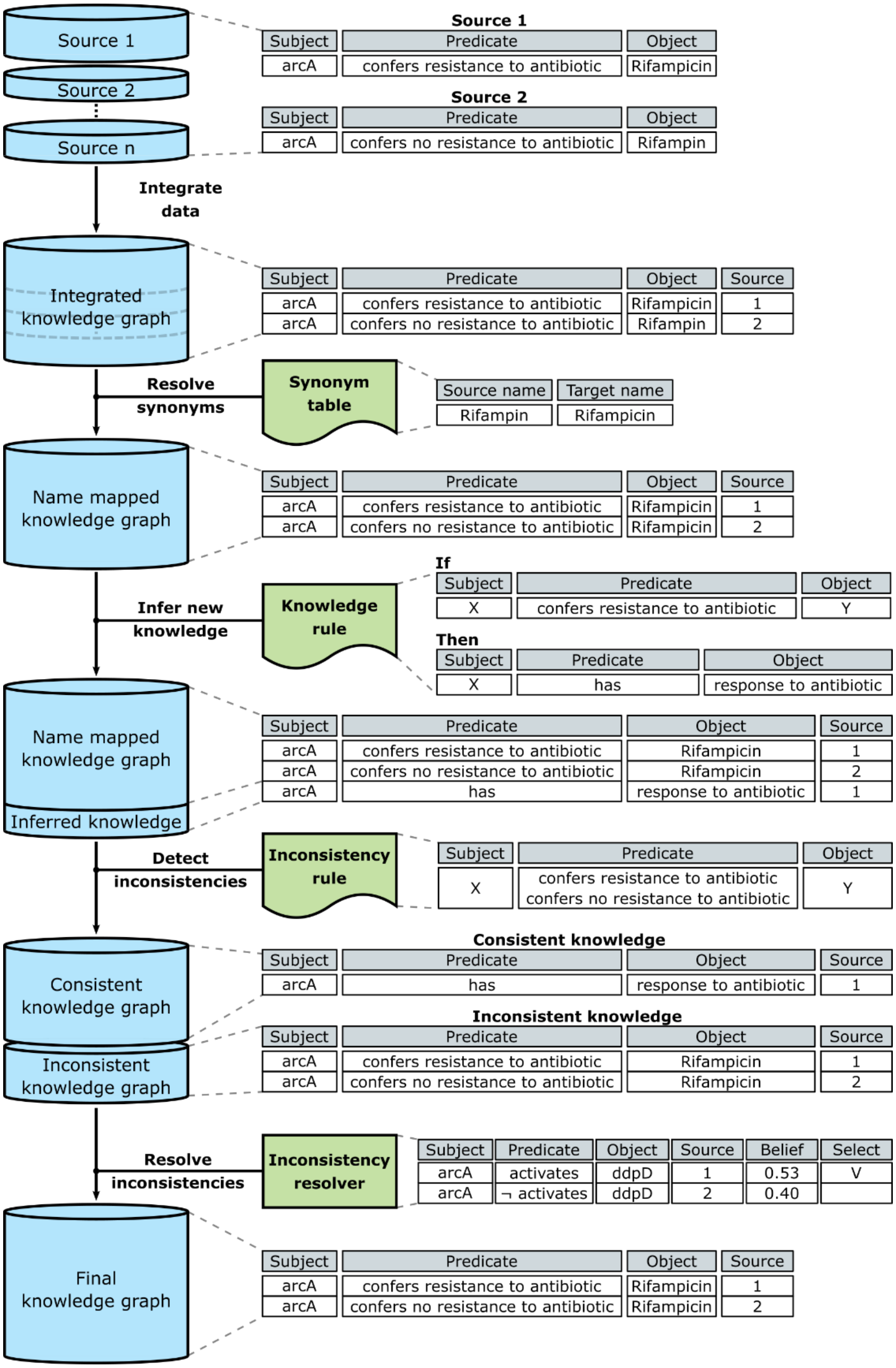

Supplementary Figure 2. Knowledge graph construction and inconsistency resolution process with examples.

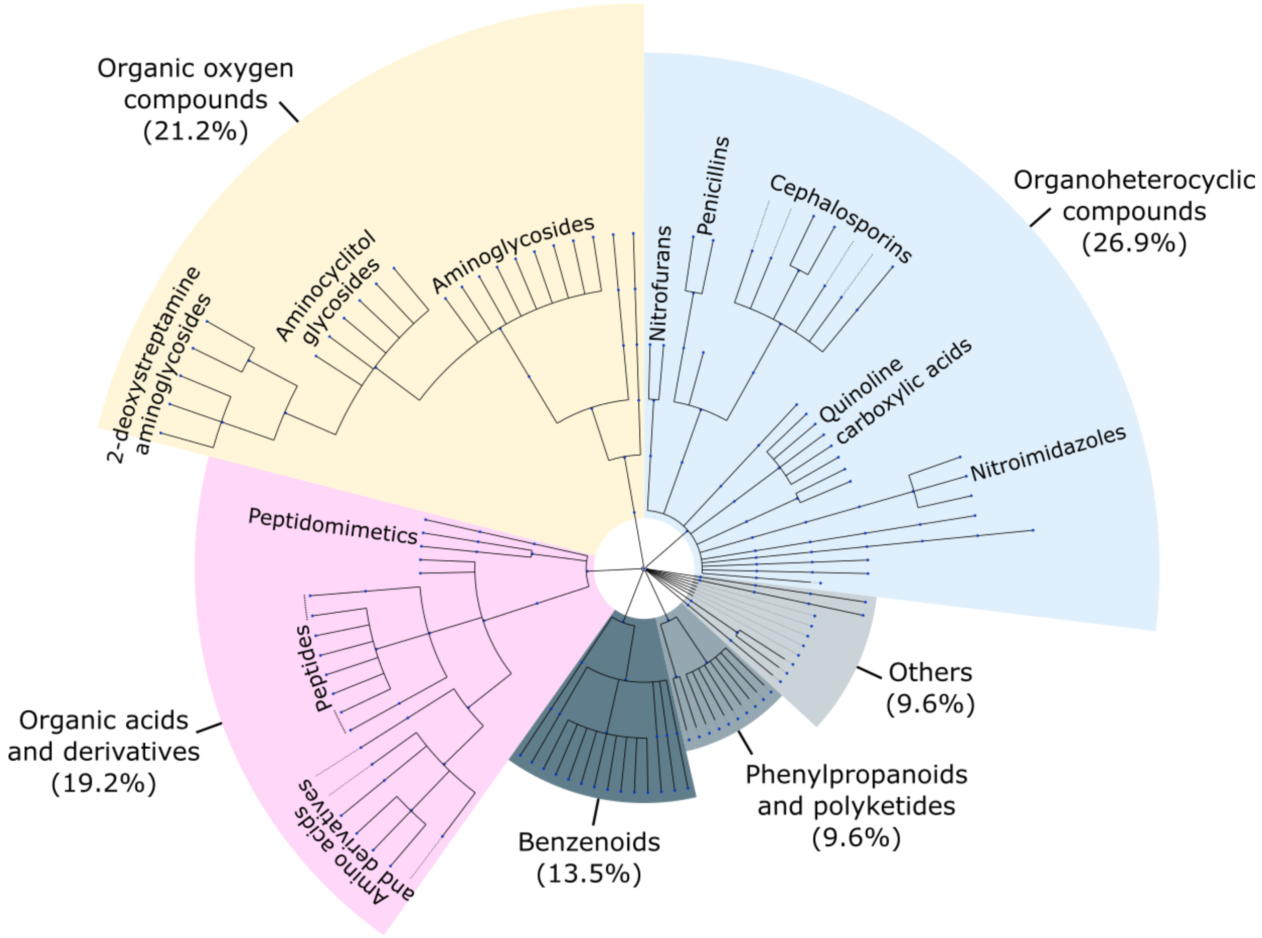

Supplementary Figure 3. Cladogram showing 6 unique taxonomic groups of antibiotics present in the knowledge graph. We classified the 104 antibiotics present in the knowledge graph into 6 taxonomic groups of antibiotics using the chemical classification ontology.

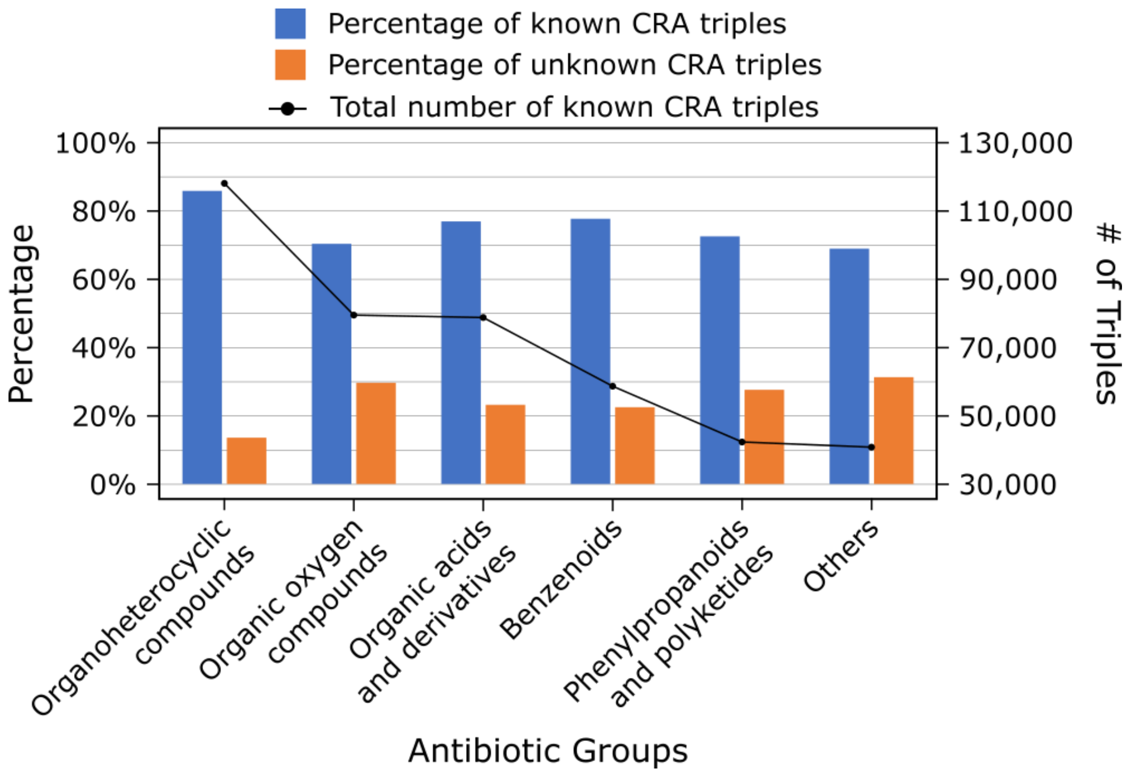

Supplementary Figure 4. The bar chart shows the percentage of known and unknown triples with CRA predicate for each taxonomic group of antibiotics. Known triples are used for training the hypothesis generator, whereas hypotheses will be generated for the unknown triples. The solid line shows the actual number of CRA triples for each group.

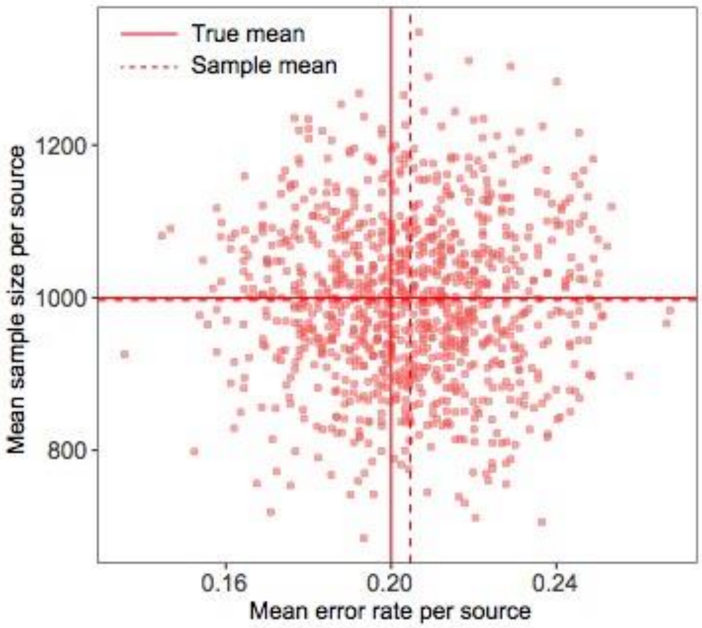

Supplementary Figure 5. Comparison of sampled mean and true mean for the two parameters (sample size per source and error rate per source) used in generating the synthetic datasets.

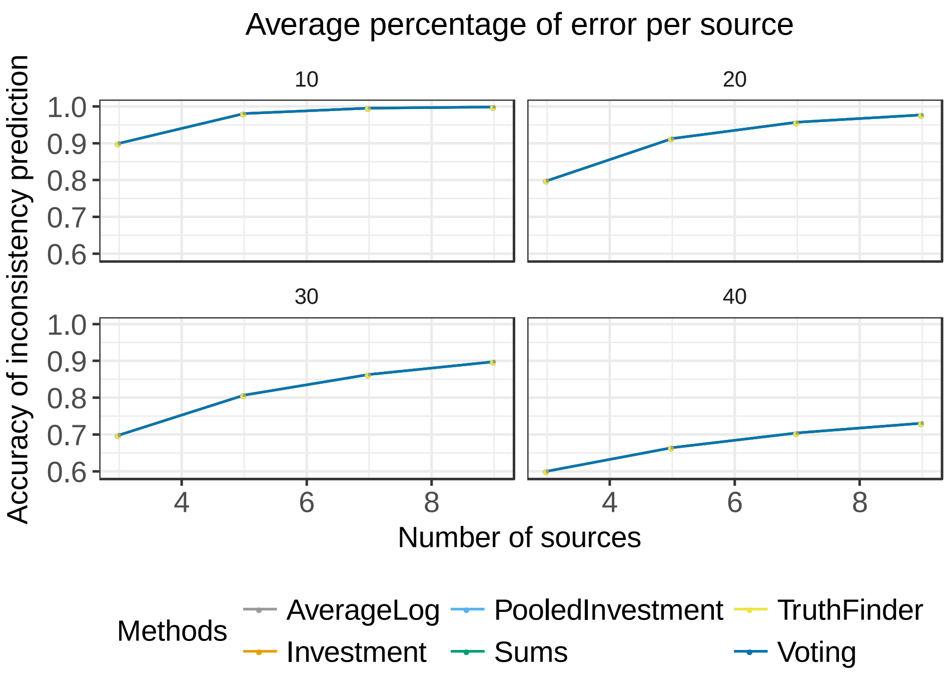

Supplementary Figure 6. Accuracy comparison of six inconsistency correction methods where the number of triples per source and error rate per source is fixed.

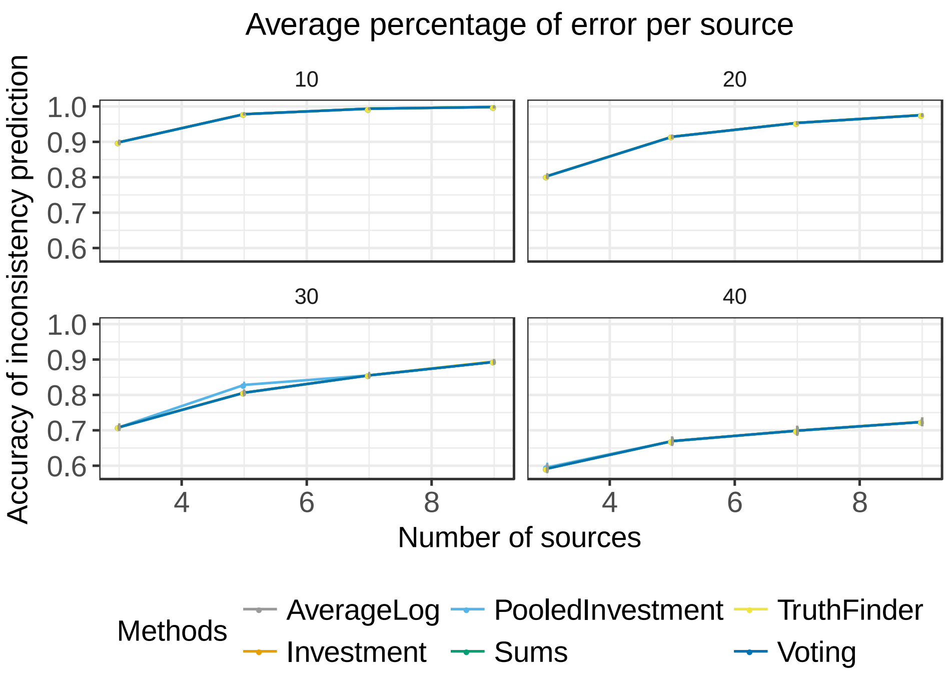

Supplementary Figure 7. Accuracy comparison of 6 inconsistency correction methods where the number of triples per source and error rate per source is sampled.

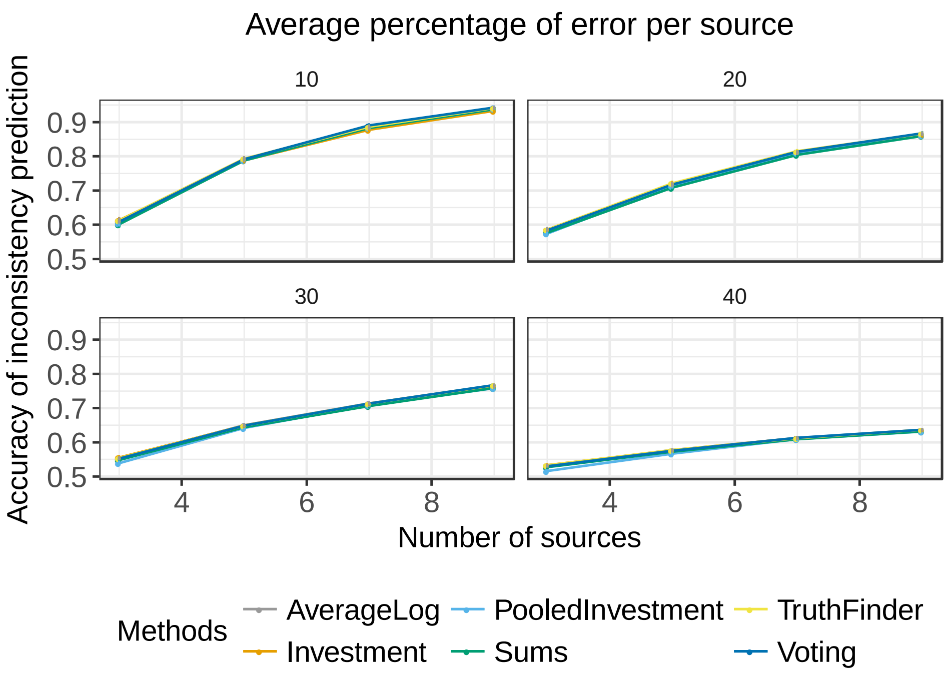

Supplementary Figure 8. Accuracy comparison of 6 inconsistency correction methods where the number of triples per source is fixed and the error rate per source is sampled.

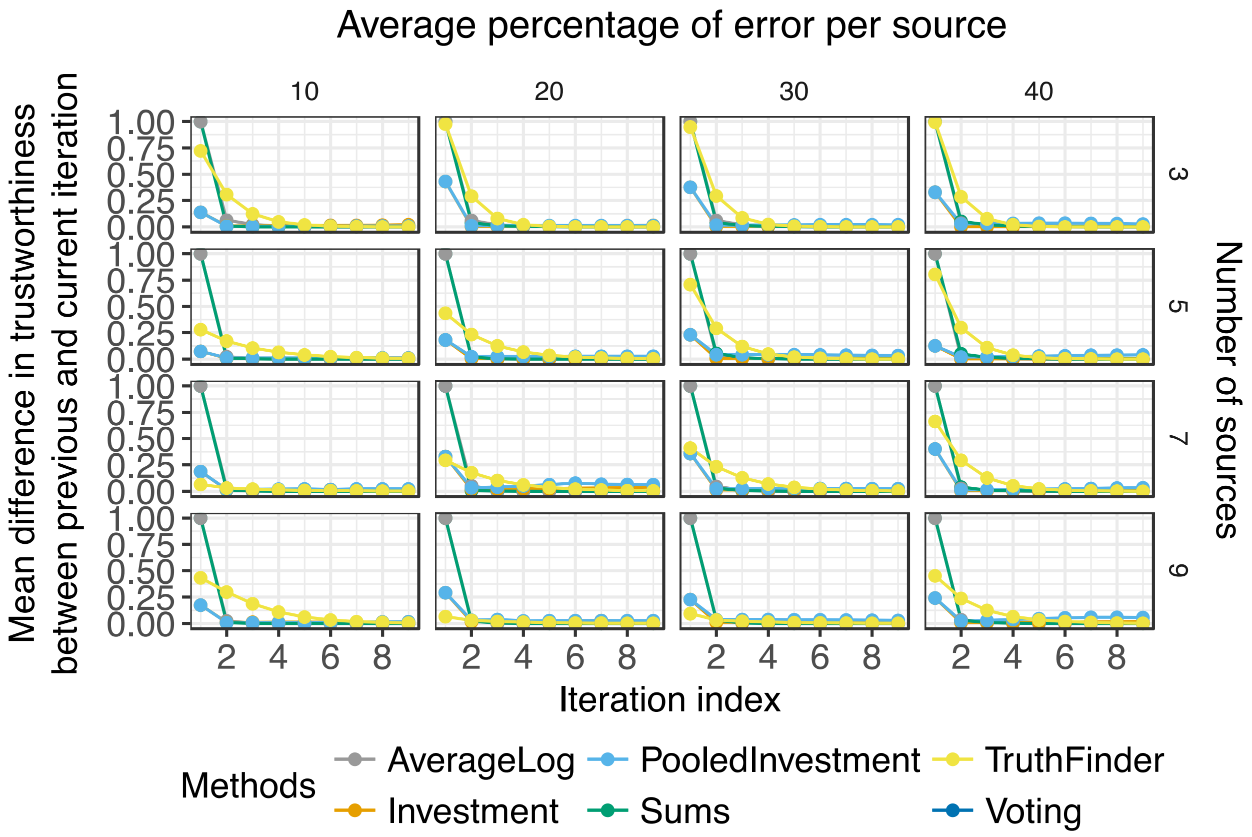

Supplementary Figure 9. The convergence of relative trustworthiness measured by inconsistency correction methods through multiple iterations.

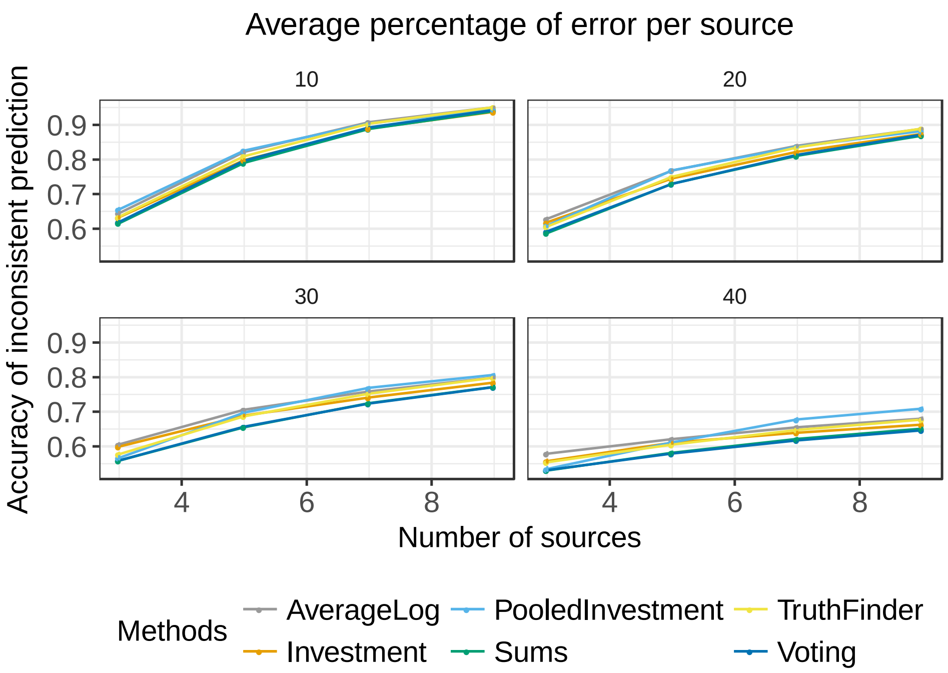

Supplementary Figure 10. Accuracy comparison of the 6 inconsistency correction methodshe number of triples per source and error rate per source is sampled.

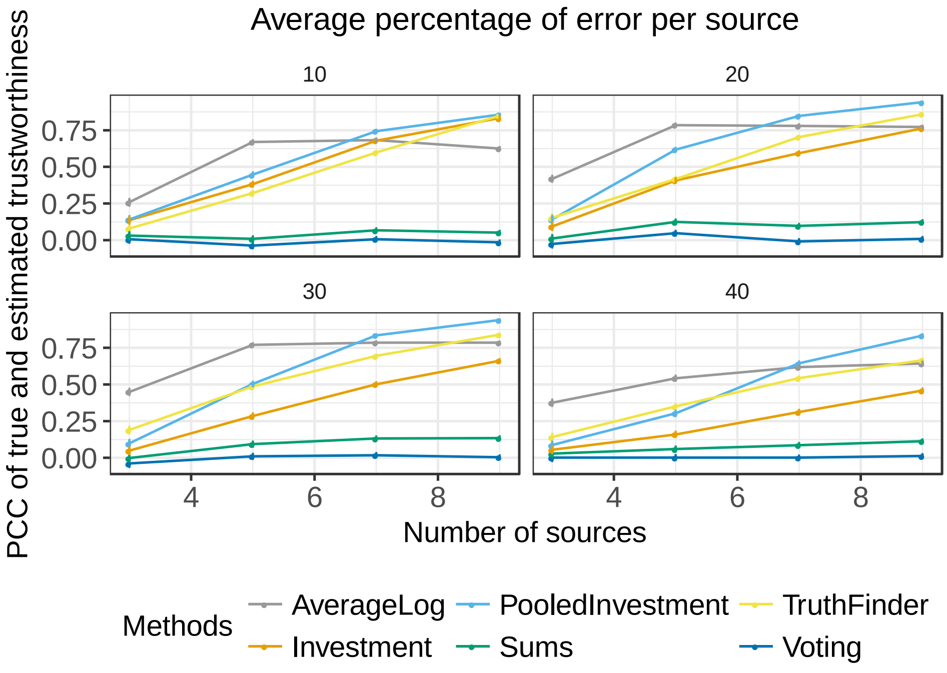

Supplementary Figure 11. The trustworthiness of sources estimated by 6 inconsistency correction methods and the ratio of true triples per source. The number of triples per source and error rate per source are sampled.

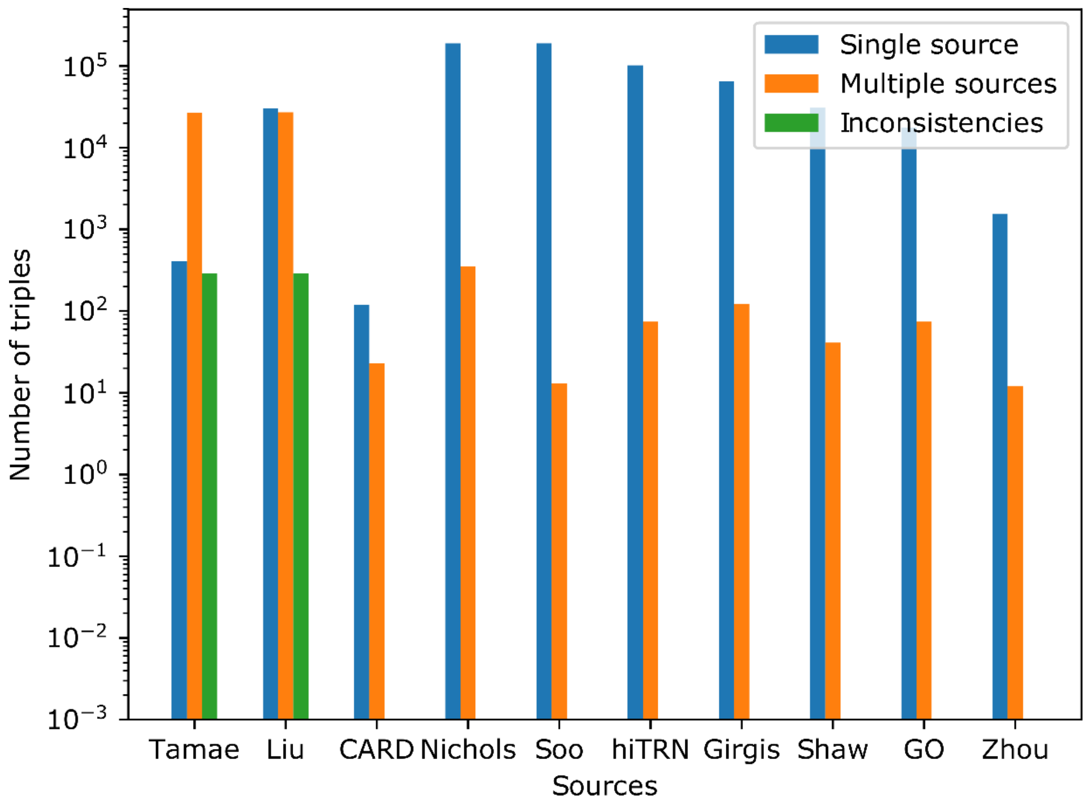

Supplementary Figure 12. The number of conflicting triples for inconsistency resolution level 1. Distribution of triples among different sources where 291 sets of inconsistencies (green) originate from Tamae and Liu between the two predicates: '*confers resistance to antibiotic after 18 hours*' and '*confers no resistance to antibiotic after 18 hours.*'

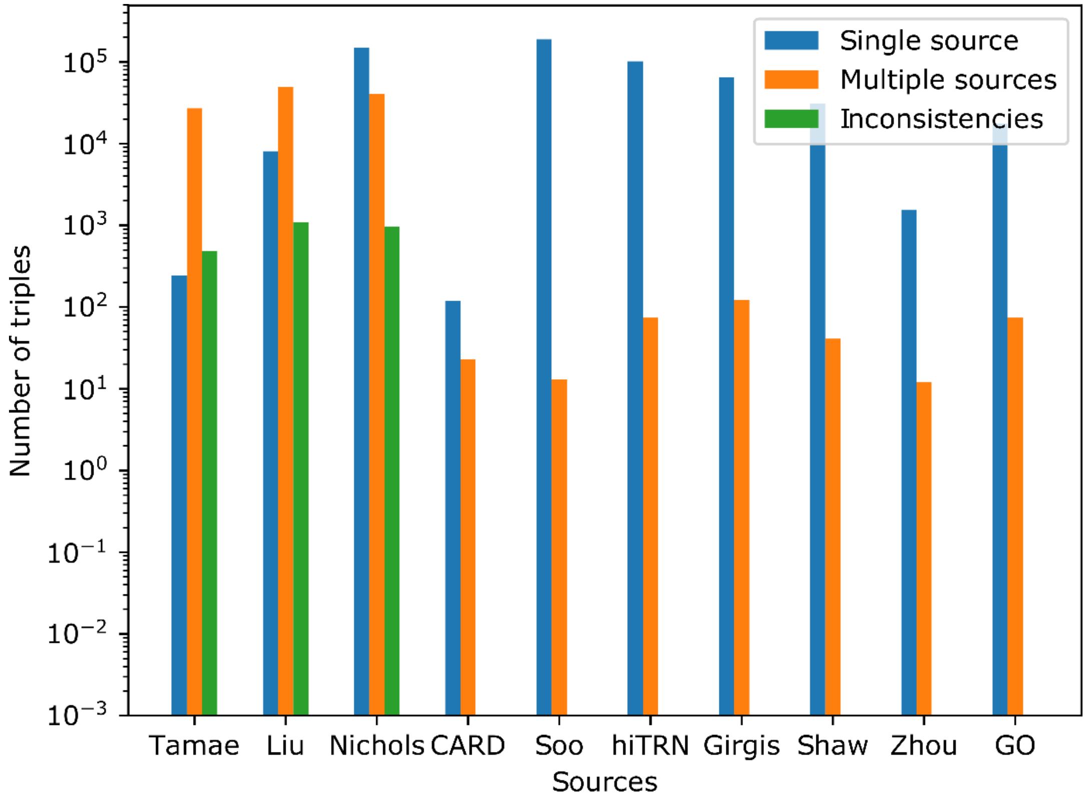

Supplementary Figure 13. The number of conflicting triples for inconsistency resolution level 2. Distribution of triples among different sources after alleviating the inconsistency detection criteria by considering antibiotic exposure time of 15 hours and 18 hours to be negligible. In this case, 1,096 sets of inconsistencies originate from Tamae et al., Liu et al., and Nichols et al. between the predicates: '*confers (no) resistance to antibiotic after 15 hours*' and '*confers (no) resistance to antibiotic after 18 hours*.'

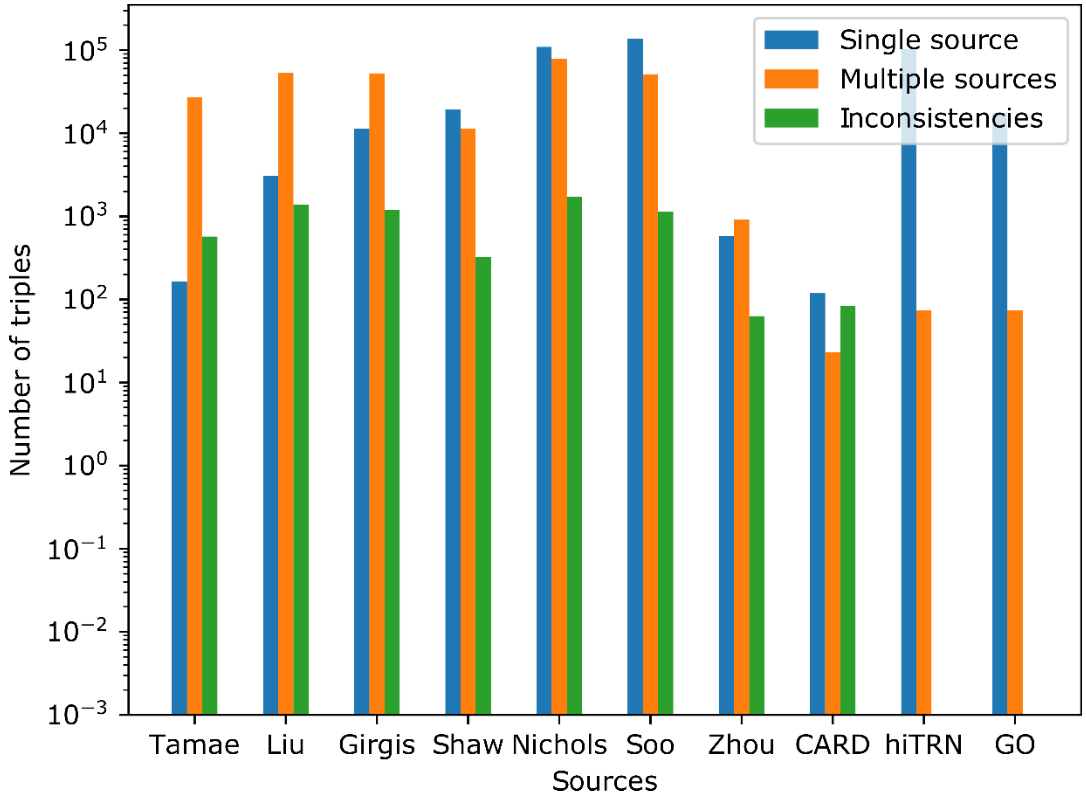

Supplementary Figure 14. The number of conflicting triples for inconsistency resolution level 3. Distribution of triples among different sources after expanding the inconsistency detection criteria to treat all CRA edges to be the same regardless of their source characteristics. In this case, 2,131 sets of inconsistencies originate from all sources but hiTRN and GO.

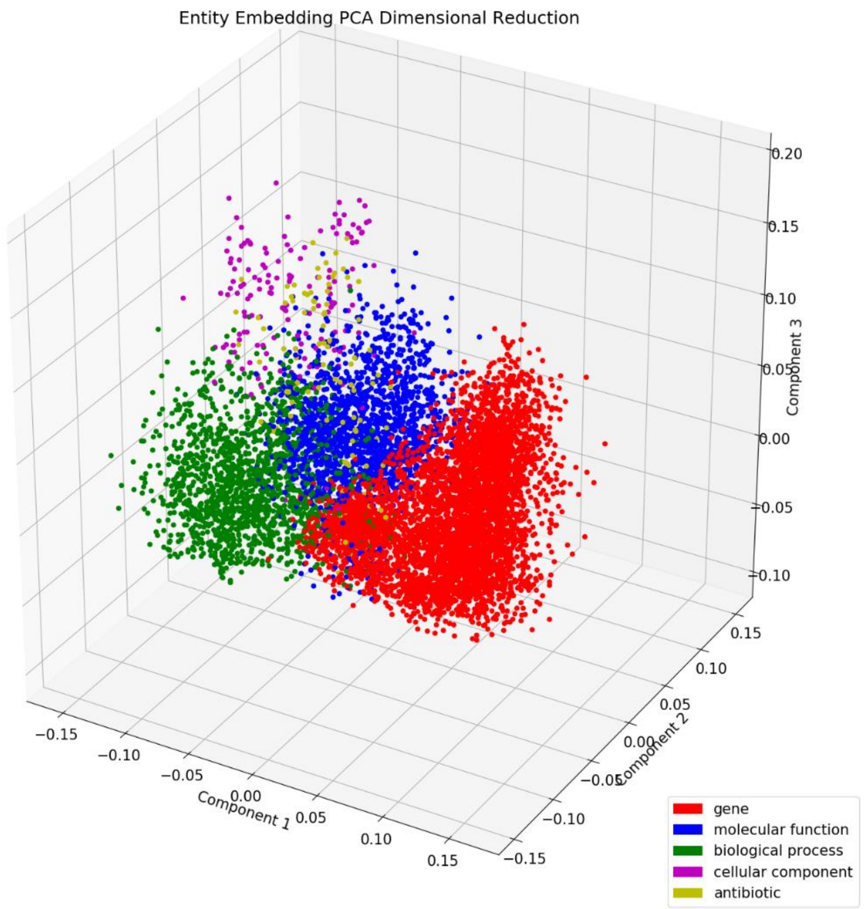

Supplementary Figure 15. Visualization of the dimensional reduction performed on the entity embeddings using principal component analysis. The initialization of these embeddings was random at the start of training; however, the noticeable clusters formed after training shows that a semantic space was produced, with each entity type predominantly in their cluster. The interesting aspect of these clusters is that the MLP was never provided the entity types during training. It simply learned the entity types based on their relationship with other entities in the knowledge graph.

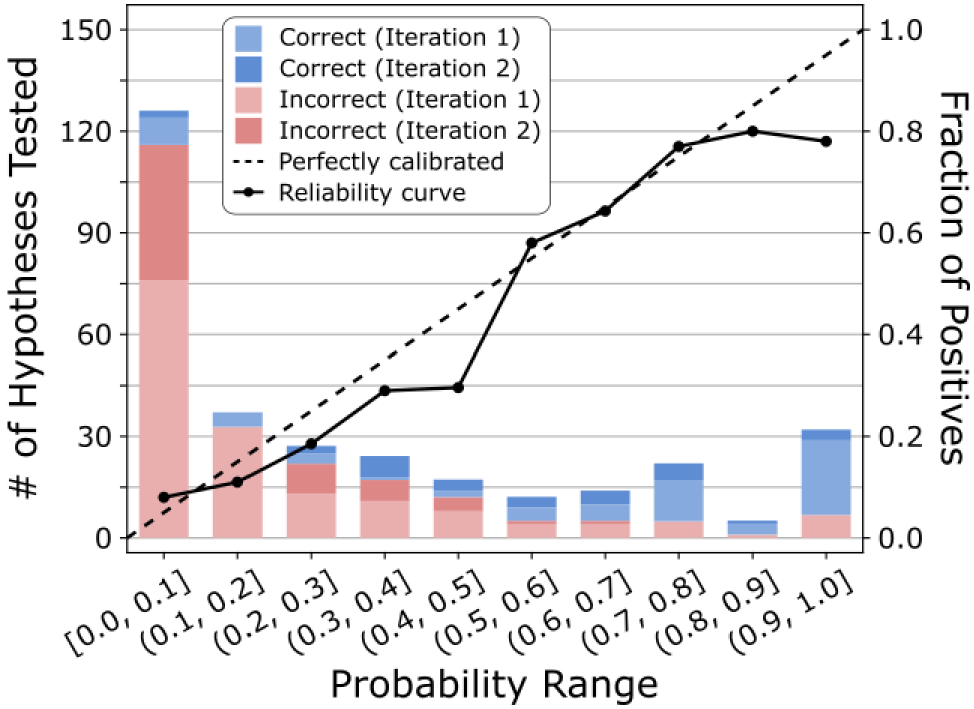

Supplementary Figure 16. Analysis of the validated hypotheses from the two iterations of hypotheses generation with 10 bins. Binning the probability of the 316 hypotheses from both iterations (226 and 90 from first and second iterations, respectively) into 10 bins shows a high correlation between the probability assignment by the hypothesis generator and forward experimental validation (R^2=0.92).

3 Supplementary Tables

Supplementary Table 1. Comparison of our *E. coli* knowledge graph with individual sources used for creating the knowledge graph. We integrated data from 10 different sources to create a comprehensive *E. coli* knowledge graph.

Source	Entity types					Knowledge discovery method	# of triples
	Gene	Antibiotic	Molecular function	Biological process	Cellular component		
CARD	72	33	-	-	-	Curation of existing knowledge	147
GO	3,672	-	1,782	1,522	152	Curation of existing knowledge	17,739
Liu et al.	3,851	22	-	-	-	MIC profile	55,877
Tamae et al.	3,850	7	-	-	-	MIC profile	26,926
Shaw et al.	3,839	4	-	-	-	Expression profile	15,327
Nichols et al.	3,666	51	-	-	-	Growth profile	186,941
Zhou et al.	47	31	-	-	-	Phenotype microarray	1,457
Soo et al.	4,294	44	-	-	-	Phenotype microarray	188,936
hiTRN	3,686	-	-	-	-	Curation of existing knowledge	101,878
Girgis et al.	3,813	17	-	-	-	Growth profile	63,636
**Total**							**658,864**
**Ours**	4,488	104	1,782	1,522	152	Curation of existing knowledge	651,758

Supplementary Table 2. The number of triples for each triple type in the knowledge graph. The domain represents the type of entities that can act as a subject node for a certain predicate, while the range represents the type of entities that can act as an object node for a certain predicate.

Domain	Predicate	Range	# of triples
gene	activates	gene	2,549
	no activates		48,312
	represses		2,473
	no represses		48,544
	upregulated by antibiotic after 30 minutes	antibiotic	100
	not upregulated by antibiotic after 30 minutes		15,227
	confers resistance to antibiotic		3,642
	confers resistance to antibiotic after 30 minutes		100
	confers resistance to antibiotic after 15 hours		1,613
	confers resistance to antibiotic after 18 hours		1,086
	confers resistance to antibiotic after 36 hours		78
	confers resistance to antibiotic after 3 days		576
	confers resistance to antibiotic after7 days		59
	confers no resistance to antibiotic after 30 minutes		15,227
	confers no resistance to antibiotic after 15 hours		185,328
	confers no resistance to antibiotic after 18 hours		54,753
	confers no resistance to antibiotic after 36 hours		1,379
	confers no resistance to antibiotic after 3 days		63,027
	confers no resistance to antibiotic after 7 days		188,745
	has	molecular function	8,115
	is involved in	biological process	6,481
	is part of	cellular component	4,313
	targeted by	antibiotic	31
**Total**			**651,758**

Supplementary Table 3. The confusion matrix of the computational inconsistency resolver for different levels of inconsistency. Wet-lab validation results are considered to be ground truth.

Level 1		Actual			
		True	False		
Predicted	True	5	30	14.3%	Precision
	False	2	199	99.9%	NPV
		71.4%	86.9%	F1	23.8%
		Recall	Specificity		

Level 2		Actual			
		True	False		
Predicted	True	7	19	26.9%	Precision
	False	0	210	100.0%	NPV
		100.0%	91.7%	F1	42.4%
		Recall	Specificity		

Level 3		Actual			
		True	False		
Predicted	True	7	14	33.3%	Precision
	False	0	215	100.0%	NPV
		100.0%	93.9%	F1	50.0%
		Recall	Specificity		

Supplementary Table 4. The number of triples for each triple type in the modified knowledge graph. We used a modified version of the knowledge graph where the temporal information was removed.

Domain	Predicate	Range	# of triples
gene	activates	gene	2,549
	no activates		48,312
	represses		2,473
	no represses		48,544
	upregulated by antibiotic	antibiotic	100
	not upregulated by antibiotic		15,227
	confers resistance to antibiotic		1,606
	confers no resistance to antibiotic		357,068
	has	molecular function	8,115
	is involved in	biological process	6,481
	is part of	cellular component	4,313
	targeted by	antibiotic	31
**Total**			**494,819**

Supplementary Table 5. The confusion matrix for different types of hypotheses generators. The entries show the average and standard deviation of the 5-fold cross-validation.

**PRA**		**Actual**			
		**True**	**False**		
**Predicted**	**True**	50.2 ± 9.2	317.0 ± 77.0	13.9% ± 1.4%	**Precision**
	**False**	269.0 ± 9.3	15323.8 ± 82.3	98.3% ± 0.1%	**NPV**
		15.7% ± 2.9%	98.0% ± 0.5%	**F1**	14.6% ± 1.2%
		**Recall**	**Specificity**		

**MLP**		**Actual**			
		**True**	**False**		
**Predicted**	**True**	100.8 ± 5.7	281.2 ± 56.5	26.9% ± 3.4%	**Precision**
	**False**	218.4 ± 5.6	15359.6 ± 48.6	98.6% ± 0.0%	**NPV**
		31.6% ± 1.8%	98.2% ± 0.4%	**F1**	28.9% ± 1.9%
		**Recall**	**Specificity**		

**Stacked**		**Actual**			
		**True**	**False**		
**Predicted**	**True**	77.6 ± 7.9	117.8 ± 24.2	40.3% ± 4.2%	**Precision**
	**False**	241.6 ± 8.2	15523.0 ± 43.2	98.5% ± 0.0%	**NPV**
		24.3% ± 2.5%	99.2% ± 0.2%	**F1**	**30.1% ± 1.7%**
		**Recall**	**Specificity**		

**TransE**		**Actual**			
		**True**	**False**		
**Predicted**	**True**	91.4 ± 10.0	436.0 ± 93.9	17.7% ± 2.0%	**Precision**
	**False**	227.8 ± 10.2	15204.8 ± 106.4	98.5% ± 0.1%	**NPV**
		28.6% ± 3.2%	97.2% ± 0.6%	**F1**	21.7% ± 1.1%
		**Recall**	**Specificity**		

**TransD**		**Actual**			
		**True**	**False**		
**Predicted**	**True**	68.6 ± 18.0	182.2 ± 54.7	27.5% ± 2.6%	**Precision**
	**False**	250.6 ± 18.3	15458.6 ± 67.2	98.4% ± 0.1%	**NPV**
		21.5% ± 5.6%	98.8% ± 0.4%	**F1**	23.7% ± 3.8%
		**Recall**	**Specificity**		

TuckER		Actual			
		True	False		
Predicted	True	97.6 ± 3.6	220.2 ± 41.2	31.1% ± 4.5%	Precision
	False	221.6 ± 3.8	15420.6 ± 43.7	98.6% ± 0.0%	NPV
		30.6% ± 1.2%	98.6% ± 0.3%	F1	30.8% ± 2.4%
		Recall	Specificity		

Supplementary Table 6. Overview of the experimental setup used to find the ARGs for each source. Source characteristics of the 7 sources that are related to ARGs are shown here.

Source	*E. coli* parent strain	Strain characteristics	Method	Media	Temperature	Length of exposure
Liu et al.	BW25113	Single knockout genes (Full Keio collection)	MIC	LB agar	37°C	18 hrs
Tamae et al.	BW25113	Single knockout genes (Full Keio collection)	MIC	LB Agar	37°C	18 hrs
Shaw et al.	MG1655	Wild type	Gene expression (microarray)	LB	37°C	30 min
Nichols et al.	BW25113	Single knockout genes (Keio collection)	Growth profile	LB agar	37°C	14-16 hrs
	BW25113	SPA-tagged derivatives of essential genes	Quantitative growth scores	LB agar	37°C	14-16 hrs
	BW25113	Point-mutants	Quantitative growth scores	LB agar	37°C	14-16 hrs
	BW25113	DAS-tagged essential genes	Quantitative growth scores	LB agar	37°C	14-16 hrs
	BW25113	Truncated genes	Quantitative growth scores	LB agar	37°C	14-16 hrs
	MG1655	Deletion of sRNA and small proteins	Quantitative growth scores	LB agar	37°C	14-16 hrs
Zhou et al.	BW25113	Deletion of two component systems	Phenotype MicroArrays	Various	36°C	24 or 48 hrs
Soo et al.	DH5α-E	Overexpression of genes with plasmid (ASKA)	Phenotype MicroArrays	IF10	37°C	up to 7 days
Girgis et al.	MG1655 ΔlacZ	Transposon-mutagenized library	Genetic footprinting	M9	37°C	2-4 days

Supplementary Table 7. Hyperparameter investigation of TruthFinder. Optimal hyperparameters are compared under the different configurations of the simulated datasets (number of sources and error rate of sources). We chose each of two TruthFinder parameters with the one that appears most often as optimal among the configurations explored. We find the performance of the chosen parameters has little difference from the performance optimized for each of the dataset configurations.

**Parameters of synthetic dataset**		**Optimal parameters of TruthFinder**		**Mean accuracy**	**Chosen parameters of TruthFinder**		**Mean Accuracy**
**Sources**	**Error rate**	ρ	γ		ρ	γ	
**3**	**0.1**	2.0	0.8	0.688	1.8	0.8	0.676
**3**	**0.4**	1.8	0.2	0.587	1.8	0.8	0.582
**9**	**0.1**	1.8	0.8	0.952	1.8	0.8	0.952
**9**	**0.4**	1.8	0.8	0.688	1.8	0.8	0.688

Supplementary Table 8. Impact of the size of knowledge sources in the accuracy of inconsistency correction (PooledInvestment). The impact is tested under the different configurations of the simulated datasets (number of sources and error rate of sources).

Parameters of the synthetic dataset			Accuracy
**# of sources**	**Error rate**	**# of triples per source**	
3	0.1	1,000	87.07
		2,000	88.62
		10,000	90.20
		20,000	90.04
	0.2	1,000	77.26
		2,000	79.68
		10,000	79.27
		20,000	80.58
	0.3	1,000	70.96
		2,000	70.44
		10,000	69.96
		20,000	70.01
	0.4	1,000	61.11
		2,000	62.11
		10,000	60.03
		20,000	59.48
5	0.1	1,000	98.28
		2,000	97.02
		10,000	97.83
		20,000	97.83
	0.2	1,000	89.88
		2,000	91.38
		10,000	91.67
		20,000	91.89
	0.3	1,000	83.00
		2,000	81.86
		10,000	80.93
		20,000	80.89
	0.4	1,000	65.30
		2,000	66.47
		10,000	66.82
		20,000	66.20

7	0.1	1,000	99.43
		2,000	99.42
		10,000	99.33
		20,000	99.43
	0.2	1,000	96.49
		2,000	95.34
		10,000	95.95
		20,000	95.79
	0.3	1,000	86.71
		2,000	87.28
		10,000	86.40
		20,000	86.33
	0.4	1,000	71.46
		2,000	69.05
		10,000	69.96
		20,000	70.32
9	0.1	1,000	100.00
		2,000	99.83
		10,000	99.82
		20,000	99.87
	0.2	1,000	97.22
		2,000	97.69
		10,000	97.65
		20,000	97.71
	0.3	1,000	88.69
		2,000	88.96
		10,000	89.51
		20,000	89.60
	0.4	1,000	73.01
		2,000	73.06
		10,000	72.69
		20,000	73.14

Supplementary Table 9. Path features used by the PRA for the first iteration of hypothesis generation. The PRA used the path features below for training a logistic regression model to infer missing edges in the knowledge graph. Weights are proportionate to the importance of the corresponding path features.

Path Feature	Weight
$gene \xrightarrow{is\ involved\ in} biological\_process \xrightarrow{is\ involved\ in^{-1}} gene \xrightarrow{confers\ resistance\ to\ antibiotic} antibiotic$	5.25
$gene \xrightarrow{has} molecular\_function \xrightarrow{has^{-1}} gene \xrightarrow{confers\ resistance\ to\ antibiotic} antibiotic$	2.75
$gene \xrightarrow{upregulated\ by\ antibiotic} antibiotic \xrightarrow{confers\ resistance\ to\ antibiotic^{-1}} gene \xrightarrow{upregulated\ by\ antibiotic} antibiotic$	2.37
$gene \xrightarrow{upregulated\ by\ antibiotic} antibiotic$	1.29
$gene \xrightarrow{upregulated\ by\ antibiotic} antibiotic \xrightarrow{confers\ resistance\ to\ antibiotic^{-1}} gene \xrightarrow{confers\ resistance\ to\ antibiotic} antibiotic$	0.74
$gene \xrightarrow{upregulated\ by\ antibiotic} antibiotic \xrightarrow{upregulated\ by\ antibiotic^{-1}} gene \xrightarrow{confers\ resistance\ to\ antibiotic} antibiotic$	0.66

Supplementary Table 10. Path features used by the PRA for the second iteration of hypothesis generation. The path features identified by the PRA vary as the knowledge graph evolves.

Path Feature	Weight
$gene \xrightarrow{is\ involved\ in} biological\_process \xrightarrow{is\ involved\ in^{-1}} gene \xrightarrow{confers\ resistance\ to\ antibiotic} antibiotic$	7.36
$gene \xrightarrow{has} molecular\_function \xrightarrow{has^{-1}} gene \xrightarrow{confers\ resistance\ to\ antibiotic} antibiotic$	4.31
$gene \xrightarrow{upregulated\ by\ antibiotic} antibiotic$	3.80
$gene \xrightarrow{upregulated\ by\ antibiotic} antibiotic \xrightarrow{confers\ resistance\ to\ antibiotic^{-1}} gene \xrightarrow{upregulated\ by\ antibiotic} antibiotic$	1.37

Supplementary Table 11. Hypothesis generator results trained on a single source. Three hypothesis generators PRA, MLP, and stacked were trained on a single source to test if the hypothesis generator trained using our knowledge graph predicts better associations than the ones trained using individual sources or the best alternative.

Sources	PRA AUCPR	MLP AUCPR	Stacked AUCPR	Comment
hiTRN	-	-	-	No CRA predicate.
GO	-	-	-	No CRA predicate.
Shaw et al.	-	-	-	No CRA predicate.
Zhou et al.	-	-	-	Not enough training data.
Nichols et al.	-	0.21±0.01	-	Cannot train PRA as these sources only contain a single CRA predicate.
Tamae et al.	-	0.38±0.07	-	
Liu et al.	-	0.46±0.04	-	
Girgis et al.	-	0.12±0.03	-	
Soo et al.	-	0.13±0.11	-	
CARD	-	-	-	Not enough training data.
Our KG	0.11±0.01	0.22±0.01	0.28±0.03	-

Supplementary Table 12. Dissemination of previously unknown ARGs across the human digestive system.

Previously unknown ARGs	# of samples containing ARG / Total # of samples	Percentage
lrp	7,292 / 94,342	7.73%
rbsK	8,062 / 94,342	8.55%
qorB	1,963 / 94,342	2.08%
hdfR	8,292 / 94,342	8.79%
ftsP	628 / 94,342	0.67%

Supplementary Table 13. PCR primers used to amplify the kanamycin cassette.

Target gene	Primer	Sequence (5→3)
lrp	Forward	ACCAGGCATTGCGCGCCGTTAATCCCTCTGGGTTTCGGTCTATCGTGATG ATTCCGGGGATCCGTCGACC
	Reverse	TCAAACTACAGCGATTTTGCACCTGTTCCGTGTTAGCGTGTCTTAATAACCAG TGTAGGCTGGAGCTGCTTCG
proV	Forward	CATGCCAGAAGCAAATTCAGGGTTGTCTCAGATTCTGAGTATGTTAGGGTATTCCGGGGATCCGTCGACC
	Reverse	CTGTGCGGTATCCCACGGATTCGTTTGATCAGCCATTGTTACCCCCCTC TGTAGGCTGGAGCTGCTTCG
rbsK	Forward	AAAGAAAAGCAGGGCACGCGCCACCCTAACCGGTGGCGCACTTTGACGTG ATTCCGGGGATCCGTCGACC
	Reverse	CGGGCAACATCTTTCATAGTAGCCAAGCGTTACCCCTGCTGATGTAAAAA TGTAGGCTGGAGCTGCTTCG
ftsP	Forward	GTTATTGTAGAAATCATTTTTTCAGGCACAACTCTTTAGCCTGTTTTACATATTCCGGGGATCCGTCGACC
	Reverse	TGCGCTATTCAGACCCGTACTCGGACGCTTTACGACGCTGGATTCACCAGTGTAGGCTGGAGCTGCTTCG
yifA	Forward	GACAGAGTGTAAAACAAAACATTTAAATCATAACGACAAATAATTTTGTGATTCCGGGGATCCGTCGACC
	Reverse	AAGTTCCCTTCTTTTTCTTTTCATCATTTTCATTGTTCATCCAGCACATCTGTAGGCTGGAGCTGCTTCG

4 References

1. Jia, B. *et al.* CARD 2017: expansion and model-centric curation of the comprehensive antibiotic resistance database. *Nucleic Acids Res.* gkw1004 (2016).

2. Consortium, G. O. Expansion of the Gene Ontology knowledgebase and resources. *Nucleic Acids Res.* **45**, D331--D338 (2016).

3. Keseler, I. M. *et al.* The EcoCyc database: reflecting new knowledge about Escherichia coli K-12. *Nucleic Acids Res.* **45**, D543--D550 (2016).

4. Liu, A. *et al.* Antibiotic sensitivity profiles determined with an Escherichia coli gene knockout collection: generating an antibiotic bar code. *Antimicrob. Agents Chemother.* **54**, 1393–1403 (2010).

5. Tamae, C. *et al.* Determination of antibiotic hypersensitivity among 4,000 single-gene-knockout mutants of Escherichia coli. *J. Bacteriol.* **190**, 5981–5988 (2008).

6. Shaw, K. J. *et al.* Comparison of the changes in global gene expression of Escherichia coli induced by four bactericidal agents. *J. Mol. Microbiol. Biotechnol.* **5**, 105–122 (2003).

7. Nichols, R. J. *et al.* Phenotypic landscape of a bacterial cell. *Cell* **144**, 143–156 (2011).

8. Baba, T. *et al.* Construction of Escherichia coli K-12 in-frame, single-gene knockout mutants: the Keio collection. *Mol. Syst. Biol.* **2**, 8–2006 (2006).

9. Zhou, L., Lei, X.-H., Bochner, B. R. & Wanner, B. L. Phenotype microarray analysis

of Escherichia coli K-12 mutants with deletions of all two-component systems. *J. Bacteriol.* **185**, 4956–4972 (2003).

10. Soo, V. W. C., Hanson-Manful, P. & Patrick, W. M. Artificial gene amplification reveals an abundance of promiscuous resistance determinants in Escherichia coli. *Proc. Natl. Acad. Sci.* **108**, 1484–1489 (2011).

11. Kitagawa, M. *et al.* Complete set of ORF clones of Escherichia coli ASKA library (A Complete S et of E. coli K-12 ORF A rchive): Unique Resources for Biological Research. *DNA Res.* **12**, 291–299 (2005).

12. Fang, X. *et al.* Global transcriptional regulatory network for Escherichia coli robustly connects gene expression to transcription factor activities. *Proc. Natl. Acad. Sci.* **114**, 10286–10291 (2017).

13. Girgis, H. S., Hottes, A. K. & Tavazoie, S. Genetic architecture of intrinsic antibiotic susceptibility. *PLoS One* **4**, e5629 (2009).

14. Bosselut, A. *et al.* Comet: Commonsense transformers for automatic knowledge graph construction. *arXiv Prepr. arXiv1906.05317* (2019).

15. Feunang, Y. D. *et al.* ClassyFire: automated chemical classification with a comprehensive, computable taxonomy. *J. Cheminform.* **8**, 61 (2016).

16. Bollacker, K., Evans, C., Paritosh, P., Sturge, T. & Taylor, J. Freebase: a collaboratively created graph database for structuring human knowledge. in *Proceedings of the 2008 ACM SIGMOD international conference on Management of data* 1247–1250 (2008).

17. Li, Y. *et al.* A survey on truth discovery. *ACM Sigkdd Explor. Newsl.* **17**, 1–16 (2016).

18. Li, X., Dong, X. L., Lyons, K., Meng, W. & Srivastava, D. Truth finding on the deep web: Is the problem solved? *Proc. VLDB Endow.* **6**, 97–108 (2012).

19. Nickel, M., Murphy, K., Tresp, V. & Gabrilovich, E. A review of relational machine learning for knowledge graphs. *Proc. IEEE* **104**, 11–33 (2016).

20. Gebser, M. *et al.* Repair and prediction (under inconsistency) in large biological networks with answer set programming. in *Twelfth International Conference on the Principles of Knowledge Representation and Reasoning* (2010).

21. Melas, I. N., Samaga, R., Alexopoulos, L. G. & Klamt, S. Detecting and removing inconsistencies between experimental data and signaling network topologies using integer linear programming on interaction graphs. *PLoS Comput. Biol.* **9**, e1003204 (2013).

22. Pasternack, J. & Roth, D. Knowing what to believe (when you already know something). in *Proceedings of the 23rd International Conference on Computational Linguistics* 877–885 (2010).

23. Kleinberg, J. M. Authoritative sources in a hyperlinked environment. *J. ACM* **46**, 604–632 (1999).

24. Yin, X., Han, J. & Philip, S. Y. Truth discovery with multiple conflicting information providers on the web. *IEEE Trans. Knowl. Data Eng.* **20**, 796–808 (2008).

25. Dingsdag, S. A. & Hunter, N. Metronidazole: an update on metabolism, structure--

cytotoxicity and resistance mechanisms. *J. Antimicrob. Chemother.* **73**, 265–279 (2018).

26. Löfmark, S., Edlund, C. & Nord, C. E. Metronidazole is still the drug of choice for treatment of anaerobic infections. *Clin. Infect. Dis.* **50**, S16--S23 (2010).

27. Dong, X. *et al.* Knowledge vault: A web-scale approach to probabilistic knowledge fusion. in *Proceedings of the 20th ACM SIGKDD international conference on Knowledge discovery and data mining* 601–610 (2014).

28. Liekens, A. M. L. *et al.* BioGraph: unsupervised biomedical knowledge discovery via automated hypothesis generation. *Genome Biol.* **12**, R57 (2011).

29. Pecina, P. Lexical association measures and collocation extraction. *Lang. Resour. Eval.* **44**, 137–158 (2010).

30. Lao, N. & Cohen, W. W. Relational retrieval using a combination of path-constrained random walks. *Mach. Learn.* **81**, 53–67 (2010).

31. Lao, N., Mitchell, T. & Cohen, W. W. Random walk inference and learning in a large scale knowledge base. in *Proceedings of the Conference on Empirical Methods in Natural Language Processing* 529–539 (2011).

32. Carlson, A. *et al.* Toward an architecture for never-ending language learning. in *Twenty-Fourth AAAI Conference on Artificial Intelligence* (2010).

33. Ding, X., Zhang, Y., Liu, T. & Duan, J. Deep learning for event-driven stock prediction. in *Twenty-Fourth International Joint Conference on Artificial Intelligence*

(2015).

34. Socher, R., Chen, D., Manning, C. D. & Ng, A. Reasoning with neural tensor networks for knowledge base completion. in *Advances in neural information processing systems* 926–934 (2013).

35. Nickel, M., Rosasco, L. & Poggio, T. Holographic embeddings of knowledge graphs. in *Thirtieth Aaai conference on artificial intelligence* (2016).

36. Bordes, A., Usunier, N., Garcia-Duran, A., Weston, J. & Yakhnenko, O. Translating embeddings for modeling multi-relational data. in *Advances in neural information processing systems* 2787–2795 (2013).

37. Wang, Z., Zhang, J., Feng, J. & Chen, Z. Knowledge graph embedding by translating on hyperplanes. in *Twenty-Eighth AAAI conference on artificial intelligence* (2014).

38. Lin, Y., Liu, Z., Sun, M., Liu, Y. & Zhu, X. Learning entity and relation embeddings for knowledge graph completion. in *Twenty-ninth AAAI conference on artificial intelligence* (2015).

39. Pearson, K. LIII. On lines and planes of closest fit to systems of points in space. *London, Edinburgh, Dublin Philos. Mag. J. Sci.* **2**, 559–572 (1901).

40. Toutanova, K. & Chen, D. Observed versus latent features for knowledge base and text inference. in *Proceedings of the 3rd Workshop on Continuous Vector Space Models and their Compositionality* 57–66 (2015).

41. Freund, Y., Schapire, R. & Abe, N. A short introduction to boosting. *Journal-Japanese Soc. Artif. Intell.* **14**, 1612 (1999).

42. Ji, G., He, S., Xu, L., Liu, K. & Zhao, J. Knowledge graph embedding via dynamic mapping matrix. in *Proceedings of the 53rd Annual Meeting of the Association for Computational Linguistics and the 7th International Joint Conference on Natural Language Processing (Volume 1: Long Papers)* 687–696 (2015).

43. Kingma, D. P. & Ba, J. Adam: A method for stochastic optimization. *arXiv Prepr. arXiv1412.6980* (2014).

44. Han, X. *et al.* Openke: An open toolkit for knowledge embedding. in *Proceedings of the 2018 conference on empirical methods in natural language processing: system demonstrations* 139–144 (2018).

45. Kazemi, S. M. & Poole, D. Simple embedding for link prediction in knowledge graphs. *arXiv Prepr. arXiv1802.04868* (2018).

46. Sun, Z., Deng, Z.-H., Nie, J.-Y. & Tang, J. Rotate: Knowledge graph embedding by relational rotation in complex space. *arXiv Prepr. arXiv1902.10197* (2019).

47. Balažević, I., Allen, C. & Hospedales, T. M. Tucker: Tensor factorization for knowledge graph completion. *arXiv Prepr. arXiv1901.09590* (2019).

48. Davis, J. & Goadrich, M. The relationship between Precision-Recall and ROC curves. in *Proceedings of the 23rd international conference on Machine learning* 233–240 (2006).

49. Kendall, M. G. A new measure of rank correlation. *Biometrika* **30**, 81–93 (1938).

50. Webber, W., Moffat, A. & Zobel, J. A similarity measure for indefinite rankings. *ACM Trans. Inf. Syst.* **28**, 1–38 (2010).
