# Supplementary material for: Knowledge integration and decision support for accelerated discovery of antibiotic resistance genes
Source: Nat Commun. 2022 Apr 29;13:2360. doi: 10.1038/s41467-022-29993-z (PMC9055065; doi:10.1038/s41467-022-29993-z)
Supplement: Supplementary file 4 — Description of Additional Supplementary Files [file 41467_2022_29993_MOESM4_ESM.pdf]

Title: Supplementary Data 1.
Description: The E. coli antibiotic resistance knowledge graph.

Title: Supplementary Data 2.
Description: Computational and wet-lab validation results of inconsistency resolution.

Title: Supplementary Data 3.
Description: List of hypotheses generated by the hypothesis generator.

Title: Supplementary Data 4.
Description: Literature search results of all positively validated hypotheses.

Title: Supplementary Data 5.
Description: Name mapping table used for preprocessing the E. coli antibiotic resistance knowledge graph.

Title: Supplementary Data 6.
Description: Knowledge inference rules.

Title: Supplementary Data 7.
Description: Inconsistency detection rules.

Title: Supplementary Data 8.
Description: Minimum inhibitory concentration (MIC) of the antibiotics used for validating hypotheses.

Title: Supplementary Data 9.
Description: Salmonella enterica single-gene knockout and minimum inhibitory concentration of the antibiotics.

Title: Supplementary Data 10.
Description: Sequence similarity of the E. coli genes to known antibiotic resistance genes in CARD.

Title: Supplementary Data 11.
Description: List of hypotheses that appeared at least once among the 100 different versions of first iteration hypotheses with probability > 0.20.
